# Pentagonal Bipyramidal First-Row Transition Metal Complexes with Macrocyclic Ligand Containing Two Pyridine‑*N*‑Oxide Pendant Arms: Structural, Magnetic, and Theoretical Studies

**DOI:** 10.1021/acs.inorgchem.5c03514

**Published:** 2025-11-10

**Authors:** Bohuslav Drahoš, Ivan Šalitroš, Radovan Herchel

**Affiliations:** † Department of Inorganic Chemistry, Faculty of Science, 48207 Palacký University Olomouc, 17. Listopadu 12, Olomouc CZ-77146, Czech Republic; ‡ Department of Inorganic Chemistry, Faculty of Chemical and Food Technology, Slovak University of Technology in Bratislava, Bratislava SK-81237, Slovakia

## Abstract

A heptadentate 15-membered pyridine-based macrocyclic ligand containing two pyridine-*N*-oxide pendant arms (**L4** = 3,12-bis­((pyridine-1-oxide-2-yl)­methyl)-6,9-dioxa-3,12,18-triazabicyclo[12.3.1]­octadeca-1(18),14,16-triene) was synthesized together with its first-row transition metal complexes with the general formula [M­(**L4**)]­(ClO_4_)_2_·1DMF (M^II^ = Mn (**1**), Fe (**2**), Co (**3**), and Ni (**4**); DMF = *N*,*N’*-dimethylformamide), which were thoroughly investigated. According to the obtained X-ray crystal structures, all complexes possess axially compressed pentagonal bipyramidal geometry with a coordination number of 7 for **1**–**3** or 5 + 2 for Ni­(II) complex **4** with a large Jahn–Teller distortion. Fe­(II), Co­(II), and Ni­(II) complexes **2**, **3**, and **4** show pronounced magnetic anisotropy (*D* = 4.47, 30.10, −7.58 cm^–1^, respectively). The magnetic properties of the studied complexes were supported by theoretical calculations, which corresponded very well to the experimental data for magnetic anisotropy. Furthermore, complex **3** showed a field-induced single-molecule magnet behavior described best by the combination of direct (*DH^m^* = 145 K^–1^s^–1^) and Raman (*C* = 0.58 K^–n^s^–1^ for *n* = 5.76) relaxation processes. Magneto-structural correlation for Fe­(II)/Co­(II)/Ni­(II) complexes with **L4** and previously studied structurally similar ligands revealed a significant impact of the coordination ability of the functional group in pendant arms on the final magnetic anisotropy (π-acceptors appear to be more suitable).

## Graphical abstract

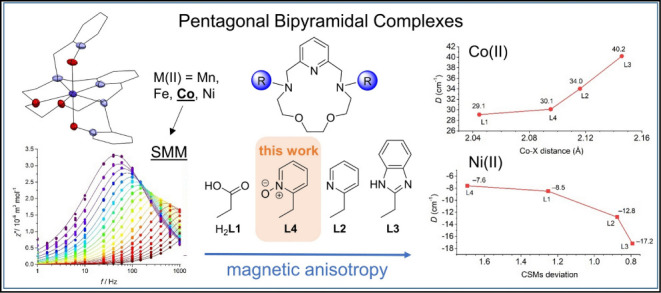

## Introduction

Seven-coordinate pentagonal bipyramidal transition metal and lanthanide complexes have gained considerable attention during the past decade, mostly due to their interesting magnetic properties.^1^ They were found to possess a large magnetic anisotropy expressed for transition metal complexes by the axial and rhombic zero-field splitting (ZFS) parameters *D* and *E*, respectively.^2^ In fact, magnetic anisotropy is the key parameter for the development of effective single-molecule magnets (SMMs), which show a slow relaxation of magnetization based on a purely molecular origin without any long-range ordering typical for bulk magnets. Thus, they can be considered as “nanomagnets” and could find many spectacular applications, e.g., high-density storage media, spintronics, or quantum computing, etc.^3^ The SMMs operate below their blocking temperatures (*T*_B_), which so far are very low. Elevating *T*_B_ requires enhancing the energy barrier for magnetic moment reversal (*U*_eff_), achieved by increasing the magnetic anisotropy of the coordinated metal centers. It was shown previously that the magnetic anisotropy could be effectively tuned by tailoring the electronic structure of the metal center by variation of the ligand field strength (different donor atoms) and/or symmetry (different coordination numbers and geometries).^4–7^

Such tuning remains highly challenging due to the limited number of established magneto-structural correlations^8^ that could elucidate how specific ligand modifications would enhance magnetic anisotropy. Therefore, such magneto-structural correlations are very valuable. Nevertheless, a breakthrough was achieved some years ago by the discovery of dysprosocenium with the blocking temperature of *T*_B_ = 60 K (*U*_eff_ = 1837 K);^9^ then, *T*_B_ even surpassed the liquid nitrogen temperature in another Dy^III^ metallocene derivative (*T*_B_ = 80 K, *U*_eff_ = 1541 cm^–1^);^10^ and today, the highest *T*_B_ has been pushed up to 100 K for a pseudolinear dysprosium bis­(amide)–alkene complex (*U*_eff_ = 1843 K).^11^

Seven-coordinate pentagonal bipyramidal complexes are not so common for the first-row transition metals.^12^ To achieve the pentagonal bipyramidal geometry of the complex, usually, a pentadentate open-chain (H_2_ **L5-R**, Figure 1) or macrocyclic (**15-pyN**_**3**_ **O**_**2**_, **L6**) ligand is coordinated in the pentagonal equatorial plane, and the two apical positions are occupied by two monovalent coligands. Previously, mainly complexes with Mn­(II),^13^ Fe­(II),^14–18^ Co­(II),^19–22^ and Ni­(II)^19,23^ metal ions have been studied,^1,24,25^ and more recently, Cr­(III)^26^ has also been investigated. Additionally, some other heavier d-metals (e.g., Mo­(IV),^27^ Mo­(III),^28^ Re­(V),^29^ and W­(III))^30^ have been investigated as well.^31^ While Co­(II) complexes usually possess positive *D*-values,^32^ suggesting easy-plane magnetic anisotropy, Fe­(II) and Ni­(II) complexes usually show negative *D*-values corresponding to the easy-axis type of magnetic anisotropy.^1^

**Figure 1:**
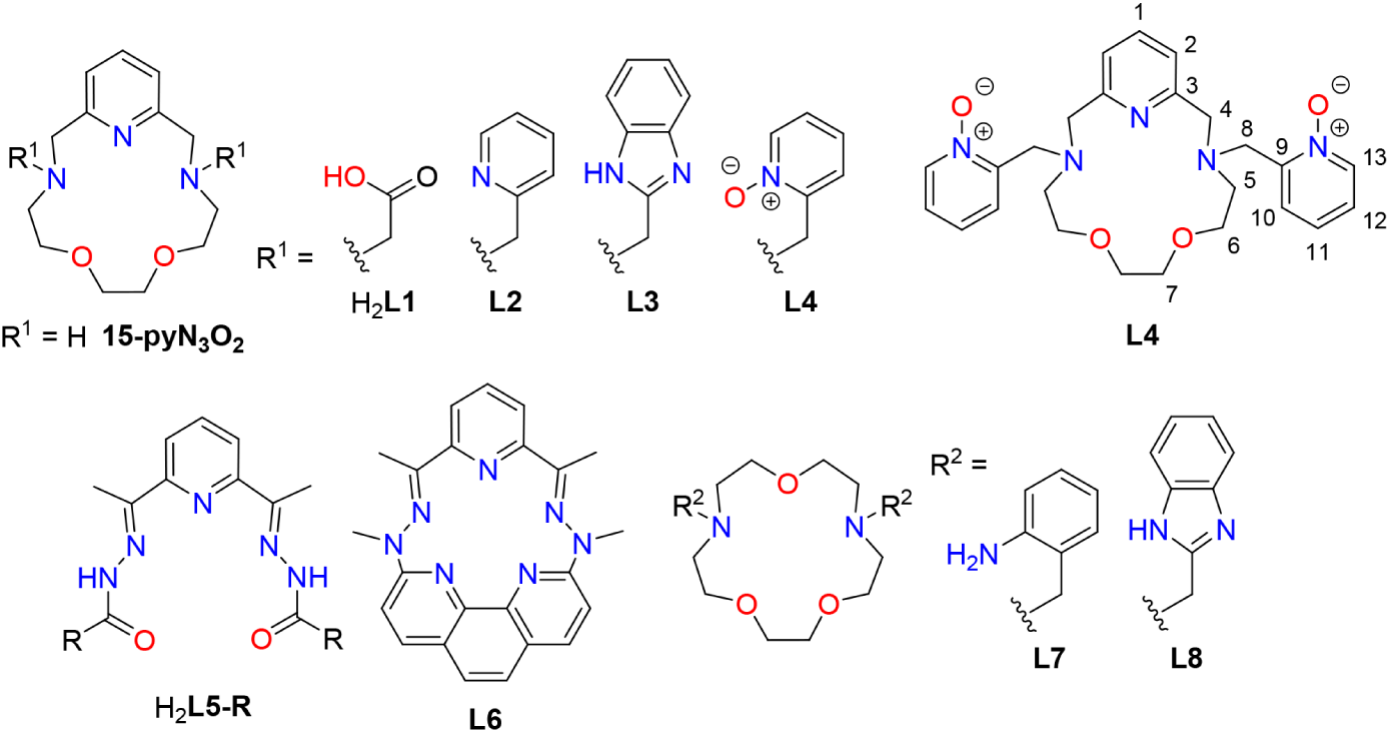
Structural formulas of the studied ligand as well as the ligands discussed in the text, together with atom numbering used in the assignment of the NMR spectra.

In the case of pentagonal bipyramidal complexes, the ligand field can be tuned by using two different approaches. The first approach is based on the exchange of small axial/apical coligands^13,21,33^ while keeping the same pentadentate ligand in the equatorial plane. The success of this strategy has been confirmed in the case of Co­(II) complexes [Co­(H_2_ **L5-Ph**)­(NCS)_2_] and [Co­(**L5-Ph)**(H_2_O)_2_];^34^ [Co­(**L6**)­(X)_2_]^2+/0^, where X = H_2_O, CN^–^, NCS^–^, SPh^–^;^21^ [Co­(H_2_ **L5-NH**_**2**_)­(X)_2_]^2+/0^, where X = SCN^–^, SeCN^–^, N­(CN)_2_^–^, C­(CN)_3_^–^, N_3_^–^;^35^ and [Co­(**15-pyN**_**3**_ **O**_**2**_)­X_2_] (X = Cl^–^, Br^–^, I^–^)^33^ and more recently also in the case of Fe­(II) complexes [Fe­(H_2_ **L5-NHPh**)­(X)_2_], where X = NCS^–^, NCSe^–^, N­(CN)_2_^–^;^36^ as well as Ni­(II) complexes [Ni­(H_2_ **L5-NH**_**2**_)­(imidazole)_2_]­(NO_3_)_2_, [Ni­(H_2_ **L5-NH**_**2**_)­(NCS)_2_], [Ni­(H_2_ **L5-Ph**)­(NCS)_2_],^37^ and [Ni­(**15-pyN**_**3**_ **O**_**2**_)­X_2_] (X = Br^–^, I^–^, CH_3_CN, NCS^–^, imidazole).^38^ The second approach for modulation of magnetic anisotropy relies on a structural modification of the basal pentadentate (usually macrocyclic) moiety with two pendant arms containing various functional groups with different coordination ability.^39,40^ Thus, the synthesis of a new heptadentate ligand with the two pendant arms coordinated in the apical position of the pentagonal bipyramid is required. This second approach has been previously rarely applied in a series of complexes with derivatives of **15-pyN**_**3**_ **O**_**2**_ containing two acetates, [M­(**L1**)],^39^ two 2-pyridylmethyl groups [M­(**L2**)]­(ClO_4_)_2_,^40^ and two 2-benzimidazolylmethyl groups [M­(**L3**)]­(ClO_4_)_2_ ^41^ (M = Mn, Fe, Co, Ni) or in Mn­(II), Co­(II), and Ni­(II) complexes with ligands **L7** ^42^ and **L8** ^43^ with 2-aminobenzyl or 2-benzimidazolylmethyl pendant arms.

In this paper, a structurally new heptadentate 15-membered macrocyclic ligand **L4** (Figure 1) containing two pyridine-*N*-oxide pendant arms has been synthesized, and its Mn­(II), Fe­(II), Co­(II), and Ni­(II) complexes were prepared and investigated. Their structural and magnetic properties were studied in detail, and the obtained results are compared to those of previously studied structurally similar macrocyclic ligands **L1**–**L3** having different functional groups in pendant arms to provide magneto-structural correlation and reveal the effect of the coordinated functional group in axial positions on the magnetic anisotropy. The obtained results are supported by theoretical calculations.

## Experimental Section

### Materials and Methods

The parent macrocycle 15-pyN_3_O_2_ (3,12,18-triaza-6,9-dioxabicyclo[12.3.1]­octadeca-1(18),14,16-triene)^40,44^ and 2-chloromethylpyridine-1-oxide^45^ were synthesized following established protocols found in the literature. All of the solvents (VWR International, Fontenay-sous-Bois, France; Lach-Ner, Neratovice, Czech Republic) and other chemicals were purchased from commercial suppliers (Acros Organics, Geel, Belgium, and Sigma-Aldrich, St. Louis, MO, USA) and were used without further purification.

^1^H and ^13^C NMR spectra were measured at laboratory temperature on a 400 MHz NMR spectrometer Varian for high-resolution solution-state NMR (Varian, Palo Alto, CA, USA). ^1^H 399.95 MHz, (CDCl_3_, tetramethylsilane) δ = 0.00 ppm, ^13^C 100.60 MHz, (CDCl_3_, residual solvent peak) δ = 77.0 ppm. Multiplicity of the signals was indicated as follows: s: singlet, d: doublet, t: triplet, quin: quintet, and m: multiplet. Deuterated solvent CDCl_3_, containing 0.03% of TMS, from Sigma-Aldrich was used as received. The atom numbering scheme used for NMR data interpretation is shown in Figure 1. The carbon and hydrogen atoms were assigned according to the spectra obtained from the two-dimensional gradient-selected (*gs*) correlation experiments ^1^H–^13^C *gs*-HMQC and ^1^H–^13^C *gs*-HMBC. Elemental analysis (C, H, N) was performed using a Flash 2000 CHNO-S analyzer (Thermo Scientific, Waltham, MA, USA). Infrared (IR) spectra of the investigated complexes were recorded on a Thermo Nicolet NEXUS 670 FT-IR spectrometer (Thermo Nicolet, Waltham, MA, USA) employing the ATR technique on a diamond plate in the range of 400–4000 cm^–1^. The mass spectra were acquired using an LCQ Fleet ion trap mass spectrometer (Thermo Scientific, Waltham, MA, USA) equipped with an electrospray ionization source and a three-dimensional ion trap detector operating in positive and negative modes. The powder X-ray diffraction (PXRD) patterns were measured on an RTG diffractometer MiniFlex600 (Rigaku, Austin, TX, USA) using Cu Kα radiation (λ = 1.5418 Å).

All herein-reported magnetic investigations were carried out on an MPMS SQUID 3 (Quantum Design Inc., San Diego, CA, USA). The exact amount of the compound was mixed with melted eicosane and filled into a gelatin capsule, which was used as the sample holder. In the case of magnetic experiments at a static magnetic DC field, the temperature dependency was recorded in the thermal range of 1.9–300 K at *B* = 0.1 T using a 1 K/min sweeping rate, and the field-dependency was measured at isothermal conditions in the range of *B* = 0–7 T. Collected data were corrected for the diamagnetism of eicosane and gelatin capsule as well as for the molecular diamagnetic contribution, which was calculated using Pascal constants. Magnetic functions were transformed into the χ*T* vs *T* and *M*_mol_ vs *B* dependencies. The experimental details about the magnetic experiments at the AC magnetic field are given in the ESI section. The ZFS parameters were assessed using the software PHI^46^ for fitting the χ_M_ *T* = f­(*T*) and *M* = f­(*H*) data.

### Crystal Data

Single crystals of all complexes **1**–**4** suitable for X-ray structure analysis were prepared by diffusion of diethyl ether vapors into the complex solution in *N*,*Ń*-dimethylformamide at 5 °C. X-ray diffraction data were collected on an XtaLAB Synergy-i (Rigaku) diffractometer equipped with a HyPix 3000 Bantam detector and a monochromatized microfocus PhotonJet-i CuK_α_ radiation source (λ = 1.54184 Å) at 100 K. The molecular structures of the studied complexes were solved by direct methods and refined by a full-matrix least-squares procedure using SHELXL in the program Olex2.^47^ The hydrogen atoms on carbon atoms were fixed into idealized positions (riding model) and assigned temperature factors of either *U*_iso_(H) = 1.2U_eq_(CH, CH_2_) or *U*_iso_(H) = 1.5U_eq_(CH_3_). The molecular and crystal structures of the studied complexes depicted in all figures were drawn using the Mercury software.^48^

### Syntheses

#### 3,12-Bis­((pyridine-1-oxide-2-yl)­methyl)-6,9-dioxa-3,12,18-triazabicyclo­[12.3.1]­octadeca-1­(18),14,16-triene **(L4)**

Parent macrocycle **15-pyN**_**3**_ **O**_**2**_ (1.625 g, 6.47 mmol), 2-chloromethylpyridine-1-oxide (1.950 g, 13.58 mmol, 2.1 eqv.), and K_2_CO_3_ (8.924 g, 26.53 mmol, 10 eqv.) were suspended in 75 mL of CH_3_CN and refluxed for 2 h. The progress of the reaction was checked by TLC (SiO_2_, CHCl_3_/MeOH 1/1) when the reactant 2-chloromethylpyridine-1-oxide (*R*_f_ = 0.8) completely disappeared. The hot suspension was filtered on a glass frit, and the filtrate was evaporated under reduced pressure to give 3.46 g of a crude brown oil. This oil was redissolved in 50 mL CHCl_3_, and the obtained solution was extracted once with 25 mL of deionized water, once with 25 mL of 5% KOH aqueous solution, and then with 50 mL of distilled water containing 3 mL of 36% HCl. The aqueous phase was extracted once with 25 mL of CHCl_3_, and then, it was neutralized by adding 25 mL of 10% aq. KOH solution (until pH 12 on pH strip), and this basic solution was extracted 4 times with 20 mL of CHCl_3_. The collected organic phases were dried with anhydrous MgSO_4_, filtered on a glass frit, and evaporated under reduced pressure. The product was obtained in the form of a dark brown crystalline solid (2.908 g, yield 96.6%). Part of the product (1.908 g) was recrystallized by dissolving it in 5 mL of MeOH, followed by the addition of 10 mL of acetone. Diffusion of diethyl ether vapors at 5 °C provided a pure light-brown crystalline product (1.351 g, yield of recrystallization 70.8%, total yield 68.4%).

MS *m*/*z* (+): 466.26 ([**L4** + H^+^]^+^, calcd. 466.25), 488.22 ([**L4** + Na^+^]^+^, calcd. 488.23), 504.18 ([**L4** + K^+^]^+^, calcd. 504.20).

^1^H NMR­(CDCl_3_): δ 2.91 (H5, t, ^3^ *J*_HH_ = 5.6 Hz, 4H), 3.51 (H7, s, 4H), 3.58 (H6, t, ^3^ *J*_HH_ = 5.6 Hz, 4H), 3.89 (H4, s, 4H), 4.16 (H8, s, 4H), 7.16 (H12, m, 2H), 7.20 (H2, d, ^3^ *J*_HH_ = 7.6 Hz, 2H), 7.29 (H11, t, ^3^ *J*_HH_ = 7.4 Hz, 2H), 7.59 (H1, t, ^3^ *J*_HH_ = 7.6 Hz, 1H), 8.18 (H10, d, ^3^ *J*_HH_ = 7.4 Hz, 2H), 8.25 (H13, d, ^3^ *J*_HH_ = 6.3 Hz, 2H).

^13^C­{^1^H} NMR (CDCl_3_): δ 54.30 (C5, C8), 61.61 (C4), 68.43 (C6), 70.38 (C7), 121.82 (C2), 123.40 (C12), 125.48 (C10), 126.09 (C11), 136.46 (C1), 139.17 (C13), 151.03 (C9), 158.35 (C3).

### General Procedure for the Preparation of Complexes **1–4**

Ligand **L4** (100 mg, 0.215 mmol, 1.1 eqv.) and an appropriate amount of M­(ClO_4_)_2_·6H_2_O (0.195 mmol, 1.0 eqv., 70 mg of Mn­(ClO_4_)_2_·6H_2_O, or 71 mg of Fe­(ClO_4_)_2_·6H_2_O, Co­(ClO_4_)_2_·6H_2_O, and Ni­(ClO_4_)_2_·6H_2_O) were dissolved in 2 mL of CH_3_CN and 3 mL of CH_3_NO_2_ and heated to reflux for 2 min. The obtained clear solution was cooled down, filtered, and left to diethyl ether vapor diffusion at 5 °C. The formation of the complex was checked by measuring MS spectra. After several days, the crystals of the desired complex were formed, isolated by filtration, and redissolved in 1 mL of DMF. The obtained solution was filtered via a Millipore syringe filter (0.45 μm). The diffusion of diethyl ether vapors into the filtrate at 5 °C resulted in the formation of well-shaped crystals, which were filtered off and dried at room temperature in air. These crystals were also suitable for X-ray diffraction analysis. **Caution!** *Although we have experienced no difficulties, perchlorate salts of metal complexes with organic ligands are potentially explosive and should be handled carefully even in small quantities.*

#### [Mn**L4**]­(ClO_4_)_2_·1DMF (**1**)

The product was isolated as lemon yellow crystals (121 mg, 78%).

MS *m*/*z* (+): 619.13 ([Mn**L4** + (ClO_4_)^−^]^+^, calcd. 619.12).

MS *m*/*z* (−): 816.90 ([Mn**L4** + 3 × (ClO_4_)^−^]^−^, calcd. 817.02), 1536.69 ([2 × (Mn**L4**) + 5 × (ClO_4_)^−^]^−^, calcd. 1537.09).

Anal. Found (Calcd) (%) for [Mn**L4**]­(ClO_4_)_2_·1DMF (C_28_H_38_Cl_2_MnN_6_O_13_, *M*_r_ = 792.48): C, 42.25 (42.44); H, 4.61 (4.83); N, 10.22 (10.60).

#### [Fe**L4**]­(ClO_4_)_2_·1DMF (**2**)

Complex **2** was prepared by the same synthetic procedure described above, but a small amount of ascorbic acid was used to prevent oxidation to Fe­(III) species. The product was isolated as bright orange crystals (105 mg, 68%).

MS *m*/*z* (+): 619.99 ([Fe**L4** + (ClO_4_)^−^]^+^, calcd. 620.12).

MS *m*/*z* (−): 817.85 ([Fe**L4** + 3 × (ClO_4_)^−^]^−^, calcd. 818.02).

Anal. Found (Calcd) (%) for [Fe**L4**]­(ClO_4_)_2_·1DMF (C_28_H_38_Cl_2_FeN_6_O_13_, *M*_r_ = 793.39): C, 42.49 (42.39); H, 4.75 (4.83); N, 10.50 (10.59).

#### [Co**L4**]­(ClO_4_)_2_·1DMF (**3**)

The product was obtained in the form of purple-pink crystals (128 mg, 82%).

MS *m*/*z* (+): 523.22 (Co**L4 –** H^+^]^+^, calcd. 523.16), 623.06 (Co**L4** + (ClO_4_)^−^]^+^, calcd. 623.12).

MS *m*/*z* (−): 820.80 ([Co**L4** + 3 × (ClO_4_)^−^]^−^, calcd. 821.02), 1546.54 ([2 × (Co**L4**) + 5 × (ClO_4_)^−^]^−^, calcd. 1545.08).

Anal. Found (Calcd) (%) for [Co**L4**]­(ClO_4_)_2_·1DMF (C_28_H_38_Cl_2_CoN_6_O_13_, *M*_r_ = 796.47): C, 42.23 (42.22); H, 4.63 (4.81); N, 10.24 (10.55).

#### [Ni**L4**]­(ClO_4_)_2_·1DMF (**4**)

The product was obtained in the form of green crystals (111 mg, yield 72%).

MS *m*/*z* (+): 522.09 (Ni**L4** – H^+^]^+^, calcd. 522.17), 622.05 (Ni**L4** + (ClO_4_)^−^]^+^, calcd. 622.12).

MS *m*/*z* (−): 819.99 ([Ni**L4** + 3 × (ClO_4_)^−^]^−^, calcd. 820.02), 1544.84 ([2 × (Ni**L4**) + 5 × (ClO_4_)^−^]^−^, calcd. 1545.08).

Anal. Found (Calcd) (%) for [Ni**L4**]­(ClO_4_)_2_·1DMF (C_28_H_38_Cl_2_NiN_6_O_13_, *M*_r_ = 796.23): C, 42.27 (42.24); H, 4.73 (4.81); N, 10.21 (10.55).

### Theoretical Methods

The ORCA software packages^49^ were utilized for theoretical computations. Specifically, the 5.0 version of ORCA was used for DFT calculations for complexes **1–4**, and the 6.1 version of ORCA was utilized for DFT calculations for metal complexes with ligands **L1–L3**. CASSCF/NEVPT2 calculations were performed with an ORCA 6.1 for all metal complexes with ligands **L1–L4**. The molecular structures of the complexes under study were extracted from the experimentally determined crystallographic data using Mercury software, in which hydrogen atom positions were normalized.^50^ A triple zeta valence quality basis set by F. E. Jorge, jorge-TZP-DKH, was used for all atoms^51^ and was exported using the Basis Set Exchange website.^52^ All auxiliary basis sets were generated by the AutoAux generation procedure.^53^ The Douglas–Kroll–Hess approach was used to treat relativistic effects.^54^ Next, state average complete active space self-consistent field (SA-CASSCF)^55^ wave functions complemented by N-electron valence second-order perturbation theory (NEVPT2)^56^ were performed. The active spaces of the CASSCF calculations were defined by metal-based d-orbitals as CAS­(*n*,5), where *n* is the number of electrons in the d-orbitals according to the electronic configuration of the given M­(II) ion. In the state-averaged approach, all multiplets and multiplicities for a given electron configuration were included. The ZFS parameters, based on dominant spin–orbit coupling contributions from excited states, were calculated through quasi-degenerate perturbation theory (QDPT),^57^ in which an approximation to the Breit–Pauli form of the spin–orbit coupling operator (SOMF approximation)^58^ and the effective Hamiltonian theory^59^ were used. Increased integration grids (defgrid3 in the ORCA convention) and tight SCF convergence criteria were used in all calculations. The *ab initio* ligand field theory (AILFT)^60,61^ was applied to calculate the energies of the d-orbitals. The calculated *D*-tensor axes and orbitals were visualized with the Chemcraft program.^62^

The DFT calculations were done with the CAM-B3LYP functional^63^ together with atom-pairwise dispersion correction (D4)^64^ with the addition of the RIJCOSX approximation.^65^ The calculated electron density was analyzed with AIMAII software.^66^

## Results and Discussion

### Syntheses and General Characterizations

The ligand was synthesized by a substitution reaction using parent macrocycle **15-pyN**_**3**_ **O**_**2**_ and 2-chloromethylpyridine-1-oxide as the alkylating agent, which was prepared according to the literature from the pyridine analogue using *m*-chloroperoxybenzoic acid^45^ in acetonitrile in the presence of K_2_CO_3_ as a base. The alkylating agent was used in slight excess (2.1 equiv) to avoid quaternary ammonium salt formation and difficulties during product separation. The progress of the reaction was monitored by TLC and by measuring the MS spectrum at the end of the reaction, containing only the disubstituted product without any signal of the parent macrocycle or monosubstituted intermediate. However, according to the NMR spectrum of the crude product, further purification was required. Several attempts using column chromatography were unsuccessful; either the product was not isolated as a pure compound or a significant loss of the product occurred. Therefore, recrystallization from a MeOH/acetone mixture by diffusion of diethyl ether vapors at 5 °C appeared as a suitable approach because the product crystallized in sufficiently pure form (the assigned 2D NMR spectra are shown in Figures S1–S3).

The synthesis of all studied complexes was based on mixing an appropriate amount of the perchlorate salt of each metal ion with **L4** in a mixture of CH_3_CN and CH_3_NO_2_. The complex formation was confirmed by measurement of MS spectra, and crystallization of the product was induced by diffusion of diethyl ether vapors at 5 °C. Initially, these complexes were prepared only in CH_3_CN, but the obtained crystals rapidly decomposed due to desolvation of one CH_3_CN molecule from the crystal solvate. Then, CH_3_NO_2_ was employed, but the same problem with the fast spontaneous desolvation of crystals remained. In the end, recrystallization from high-boiling solvents such as DMF was performed, and the obtained crystal solvates were stable (except for Ni­(II) complex **4**, which lost its DMF solvate molecule very slowly). The “first” crystallization from the CH_3_CN/CH_3_NO_2_ mixture cannot be avoided because of the removal of minor impurities coming from the ligand.

All complexes were thoroughly characterized by elemental analysis, IR spectroscopy (Figure S4), and mass spectrometry. The results of elemental analysis are in excellent agreement with the calculated values corresponding to the particular complex composition, including one DMF solvate molecule (differences between the calculated and found values are below the standard limit 0.4%). All measured IR spectra (Figure S4) show a similar pattern and contain the signals of the ligand (∼3080, ∼2935 and 2890 cm^–1^ corresponding to the aromatic CH stretching vibrations and the CH_2_ stretching vibrations; ∼1600 and ∼1450 cm^–1^ corresponding to the aromatic C=C and C=N vibrations), as well as the signals at ∼1070 cm^–1^ and ∼620 cm^–1^, corresponding to the vibrations of the perchlorate anions, and a strong signal at ∼1665 cm^–1^, corresponding to the stretching vibration of the C=O group, which confirmed the presence of the solvated DMF molecule.

The phase purity of all studied complexes **1**–**4** (prepared as very fine crystalline products without any indication of the presence of any amorphous phase) was confirmed by the measurement of PXRD patterns, which are shown in Figure S5 in ESI, and by their comparison with the diffraction data simulated from the single-crystal X-ray diffraction measurements. An excellent agreement was found between both data sets (Figure S5).

### Crystal Structure and QT-AIM Analysis

Molecular structures of all studied complexes **1**–**4** were determined by X-ray diffraction analysis, and their complex cations are shown in Figures 2 and S6. The crystal data and structure refinements for all compounds are given in Table S1.

**Figure 2:**
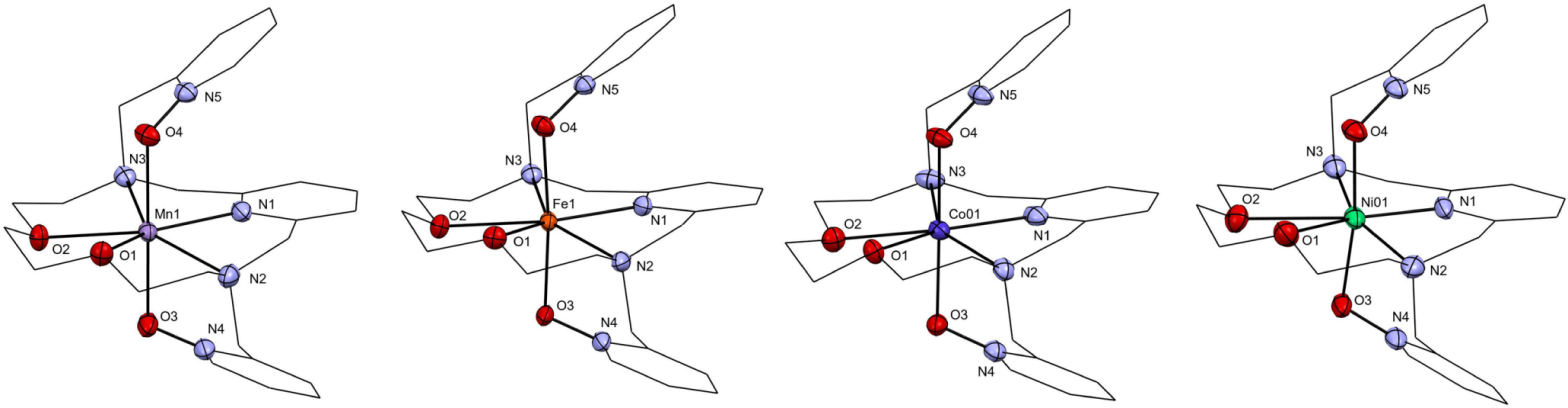
Molecular structures of the complex cations [M­(**L4**)]^2+^ of complexes **1** (Mn­(II)), **2** (Fe­(II)), **3** (Co­(II)), and **4** (Ni­(II)). Nonhydrogen atoms are drawn as thermal ellipsoids at the 50% probability level, hydrogen atoms are omitted for clarity, and carbon atoms are drawn using a wireframe style for clarity.

All the complexes **1**–**4** are isostructural; they all crystallized in the monoclinic I2/n or C2/*c* space group, and their molecular structures are similar. The charge of the complex cation, shown in Figure 2, is compensated by two perchlorate anions (at least one is disordered over two positions in each case **1**–**4**, but for complex **4**, both of them are disordered), and one solvate DMF molecule is found in the asymmetric unit (Figure S7).

The macrocyclic part of the ligand is coordinated in the equatorial pentagonal plane, while the two pyridine-*N*-oxide pendant arms occupy both axial positions. Thus, all of the complexes are seven-coordinate with pentagonal bipyramidal geometry, and the ligand **L4** is heptadentate, providing an N_3_O_4_ donor atom set. The comparison of metal–donor atom distances is shown in Figure 3, and their exact values are listed in Table 1 together with selected bond angles. The M–O3/4 (pyridine-*N*-oxide pendant arms) distances are the shortest (∼2.1 Å) for complexes **1**–**3**, and the M–N1 (macrocyclic pyridine) distance is only ∼0.1 Å longer, while in complex **4**, the situation is switched, and the shortest distance is M–N1 (∼2.0 Å). The other M–N2/3 (secondary amine) distances are ca. 0.15 Å longer, as well as the M–O1/2 (macrocyclic oxygen) distances (Figure 3). Because the axial M–O3/4 distances are much shorter than the distances in the equatorial pentagonal plane (except for M–N_py_), the pentagonal bipyramid can be described as being axially compressed. Furthermore, in the order **1** → **4** going from the Mn­(II) to Ni­(II) complex, all M–N distances decrease monotonically (Figure 3), which is in agreement with the decreasing ionic radius of chelated metal ions. The same trend can be observed for M–O3/4 distances. On the other hand, M–O1/2 distances slightly increase in the order **1** → **3** and significantly increase in the case of Ni­(II) complex **4** (Figure 3) due to a strong Jahn–Teller effect,^12^ which is typical for structurally similar seven-coordinate pentagonal bipyramidal Ni­(II) complexes, e.g., [Ni**(15-pyN**_**3**_ **O**_**2**_)­Cl_2_],^24^ [Ni**L1**],^39^ [Ni**L2**]­(ClO_4_)_2_,^40^ [Ni**L3**]­(ClO_4_)_2_,^41^ or [Ni**L7**]­(ClO_4_)_2_ ^42^ for which the Ni–O distances often exceed 2.5 Å. Thus, the pentagonal bipyramidal geometry of the Ni­(II) center in **4** is the most distorted (Ni–O distances are 2.536(2) and 2.463(2) Å; see Table 1 in bold and Figure 3), which is in agreement with the smallest O1–M–O2 angle in the series. Thus, based on these values, both oxygen atoms O1 and O2 in complex **4** can be considered as semicoordinated, and the coordination number can be regarded as 5 + 2.

**Table 1:** Selected Interatomic Distances [Å] and Angles [°] in the Studied Compounds **1**–**4** Complemented by the |*V*(**r**)|/*G*(**r**) Ratio from QT-AIM Analysis

	1-Mn	2-Fe	3-Co	4-Ni
Distances, |*V*(**r**)|/*G*(**r**)				
M–N1	2.2262(15)	2.172(2)	2.1382(18)	1.999(2)
	1.258	1.167	1.176	1.178
M–N2	2.3395(15)	2.290(2)	2.2604(17)	2.206(2)
	1.292	1.206	1.169	1.187
M–N3	2.3615(15)	2.313(2)	2.2780(17)	2.186(2)
	1.291	1.207	1.170	1.189
M–O1	2.2762( 12)	2.2806(17)	2.3007(14)	**2.5364(19)**
	1.207	1.104	1.077	1.057
M–O2	2.2739(12)	2.2919(17)	2.2878(14)	**2.4629(18)**
	1.211	1.101	1.070	1.073
M–O3	2.1648(12)	2.1093(18)	2.1042(14)	2.0417(17)
	1.220	1.147	1.138	1.139
M–O4	2.1507(12)	2.1015(17)	2.0871(14)	2.0296(16)
	1.218	1.145	1.133	1.143
Angles				
N1–M–N2	73.03(6)	74.01(8)	74.61(7)	78.79(9)
N1–M–N3	72.23(5)	73.29(8)	74.15(7)	79.04(9)
N2–M–O1	73.56(5)	72.78(7)	72.96(6)	70.69(7)
N3–M–O2	74.29(5)	73.51(7)	73.42(5)	72.62(7)
O1–M–O2	71.06(4)	70.14(6)	68.91(5)	63.34(6)
O3–M–O4	174.66(5)	174.14(7)	175.29(6)	172.16(7)

**Figure 3:**
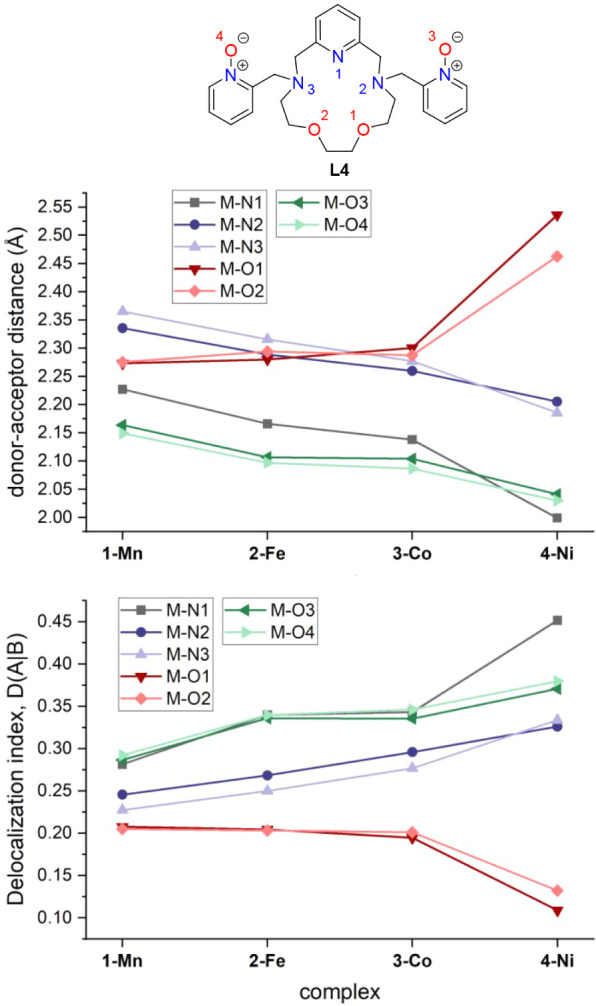
Top: comparison of the metal–donor atom distances in the studied complexes **1**–**4** depending on the type of central metal atom. Bottom: the delocalization index calculated from DFT calculations and subsequent QT-AIM analysis. The numbering of donor atoms is shown in the structural formula at the very top for the sake of clarity.

To better assess coordination bonds in reported complexes **1–4**, the quantum theory of atoms in molecules, QT-AIM,^67,68^ was employed in which the respective wave functions were calculated with the CAM-B3LYP range-separated hybrid functional using ORCA. With the help of the AIMAII software, the critical points of the type (3, −1), so-called bond critical points (BCPs), were located by the analysis of electron density ρ­(**r**). At BCPs, several functions were calculated, namely the Laplacian of the electron density [∇^2^ρ­(**r**)], electron kinetic energy [*G*(**r**)], potential energy density [*V*(**r**)], electron energy density [*h*_e_(**r**)], and bond ellipticity (ε_BCP_), which quantify the deviation of the bond from cylindrical symmetry typical for ideal single or triple bonds due to its double-bond character, mechanical strain, and other perturbations. The results are summarized in Tables 1 and S2. All donor–acceptor bonds are characterized by ∇^2^ρ­(**r**) > 0, *h*_e_(**r**) < 0, and |*V*(**r**)|/*G*(**r**) > 1; thus, the QT-AIM criteria for the definition of a coordination bond are fulfilled.^69^ The |*V*(**r**)|/*G*(**r**) ratio adopts values in the range from 1.13 to 1.29 for M–N1, M–N2, M–N3, M–O3, and M–O4 bonds in all complexes **1–4**. However, there is an evident decrease in the |*V*(**r**)|/*G*(**r**) ratio for M–O1 and M–O2 bonds from **1** to **4**, spanning the interval from 1.21 to 1.06 (Table 1). Thus, it is evident that the coordination character of M–O1/O2 bonds is weakening toward semicoordination for which |*V*(**r**)|/*G*(**r**) < 1 is expected. Moreover, the coordination bonds were characterized by calculating the AIM delocalization index (DI),^70^ which provides a quantitative measure of the electron sharing between atoms. Usually, the DI adopts a value close to 1 for a single covalent bond. The results are given in Table S2 and depicted in Figure 3. There is a steady increase in DI in the row from **1** to **4** for the M–N and M–O3/O4 bonds. However, DI of M–O1/O2 bonds is almost the same for **1–3**, and an abrupt descent of these DI is observed for **4**. This indeed suggests the transformation from the coordination type of bond to a very weak bond approaching semicoordination.

Additional analysis of the coordination polyhedra geometries of all complexes **1**–**4** was based on a comparison of continuous shape measures (CSMs, deviation between the real and ideal geometry of the polyhedron) obtained by the program Shape 2.1.^71,72^ Among the seven possible geometries for the coordination number of seven, the lowest deviation values were obtained for the pentagonal bipyramidal arrangement (Table S3). The lowest deviation was found for Fe­(II) complex **2**; thus, this complex appears to be the most symmetrical one; then, it increased for Co­(II) and Mn­(II) complexes **3** and **1**, and it was the highest for the Ni­(II) complex **4**, which is the least symmetrical one (Table 2), and this finding is in accordance with the largest M–O1/2 distances in complex **4** discussed in the previous section. Moreover, in order to investigate the symmetry of the equatorial coordination plane in pentagonal bipyramidal geometry, the CSMs for coordination number five, using the {MN_3_O_2_} chromophore with only the donor atoms of the macrocycle, were calculated as well (Table S3). The deviation from the ideal pentagon or the extent of distortion increases in the order **2** < **3** < **1** < **4**, which means that complex **2** has the most symmetrical equatorial coordination sphere (Table 2). This trend is the same when deviations from the ideal pentagonal bipyramid are compared (Tables 2 and S3). It is worth to mention that the aromatic ring of each pyridine-*N*-oxide pendant arm is turned in the complex cation unit toward the macrocyclic pyridine ring, with centroid···centroid *C*_g_···*C*_g_ distances ranging 3.626–3.690 Å in all complexes (*C*_g_···*C*_g_ = 3.635/3.672 Å (**1**), 3.626/3.653 Å (**2**), 3.626/3.662 Å (**3**), 3.635/3.690 Å (**4**)), suggesting weak π–π stacking interaction.

**Table 2:** Results of Continuous Shape Measure (CMS) Calculations Using Program Shape 2.1 for Compounds **1**–4 and Structurally Similar Complexes with Ligands **L1**, **L2,** and **L3**

	**1**-Mn(II)	**2**-Fe(II)	**3**-Co(II)	**4**-Ni(II)
CN = 5, pentagon	1.291	1.157	1.277	1.694
CN = 7, pentagonal bipyramid	1.096	1.012	1.097	1.650

	Mn(II)(**L1**)	Fe(II)(**L1**)	Co(II)(**L1**)	Ni(II)(**L1**)
CN = 5, pentagon	–	0.830	0.837	1.252
CN = 7, pentagonal bipyramid	–	3.238	3.076	2.988

	Mn(II)(**L2**)	Fe(II)(**L2**)	Co(II)(**L2**)	Ni(II)(**L2**)
CN = 5, pentagon	0.756/0.847	0.655/0.620	0.589/0.554	0.875
CN = 7, pentagonal bipyramid	1.386/1.322	1.237/1.156	0.941/0.941	1.237

	Mn(II)(**L3**)	Fe(II)(**L3**)	Co(II)(**L3**)	Ni(II)(**L3**)
CN = 5, pentagon	1.007/0.929	0.842/0.786	0.758/0.709	0.815/0.768
CN = 7, pentagonal bipyramid	1.653/1.544	1.482/1.398	1.208/1.131	1.201/1.112

### Comparison of the Obtained Molecular Structures with Those of Previously Studied Complexes with **L1–L3**

The molecular structure of metal complexes is crucial for understanding their magnetic properties, particularly the origin of their magnetic anisotropy (see the section Magnetic analysis). Thus, it is essential to provide a comparison of the obtained molecular structures of complexes **1**–**4** with those of the previously studied structurally similar ligands **L1–L3** in order to reveal any trends and correlations. While comparing metal-donor atom distances (for three sets of four complexes) would be problematic and not very clear, we decided to use the CSMs for pentagonal bipyramid and pentagon geometry in order to quantify the deviation of the coordination sphere (overall = pentagonal bipyramid or only equatorial pentagonal plane = pentagon, Table 2).

Comparing the CSM deviations for CN = 7 and pentagonal bipyramidal geometry, complexes with the studied ligand **L4** are the least distorted (except for the Co­(II) complex with **L2** and Ni­(II) complexes with **L2** and **L3**). On the other hand, these complexes **1**–**4** have the most distorted pentagonal equatorial plane, as they show the highest CSM deviations for CN = 5 and pentagonal geometry (Table 2). Thus, the distortion of the pentagonal bipyramidal geometry of the compared Mn­(II) and Fe­(II) complexes increases in the ligand order **L4** < **L2** < **L3** < **L1**. For Co­(II) complexes, the order is similar, and only **L2** and **L4** are switched, but for Ni­(II) complexes, it is **L3** < **L2** < **L4** < **L1**. On the other hand, the distortion of the equatorial pentagonal plane increases in the ligand order **L2** < **L3** < **L1** < **L4** for all complexes except Ni­(II) compounds, for which only **L2** and **L3** are switched; thus, **L3** < **L2** < **L1** < **L4** (Table 2).

### Magnetic Analysis

#### Static Magnetic Measurements

The temperature- and field-dependent experimental magnetic data for **1–4** are depicted in Figure 4. The room temperature χ_M_ *T* product value for complex **1** is close to the theoretical value (χ_M_ *T* = 4.38 cm^3^Kmol^–1^ for *S* = 2.5 (Mn­(II))), but for compelxes **2**–**4,** these values are higher than the theoretical ones for the high-spin divalent metals (χ_M_ *T* = 3.00 cm^3^Kmol^–1^ for *S* = 2 (Fe­(II)), χ_M_ *T* = 1.88 cm^3^Kmol^–1^ for *S* = 3/2 (Co­(II)), and χ_M_ *T* = 1.00 cm^3^Kmol^–1^ for *S* = 1 (Ni­(II)), all calculated with *g* = 2.0), which indicates a non-negligible contribution of the spin–orbit coupling to the ground state, and thus, *g* > 2.0.

**Figure 4:**
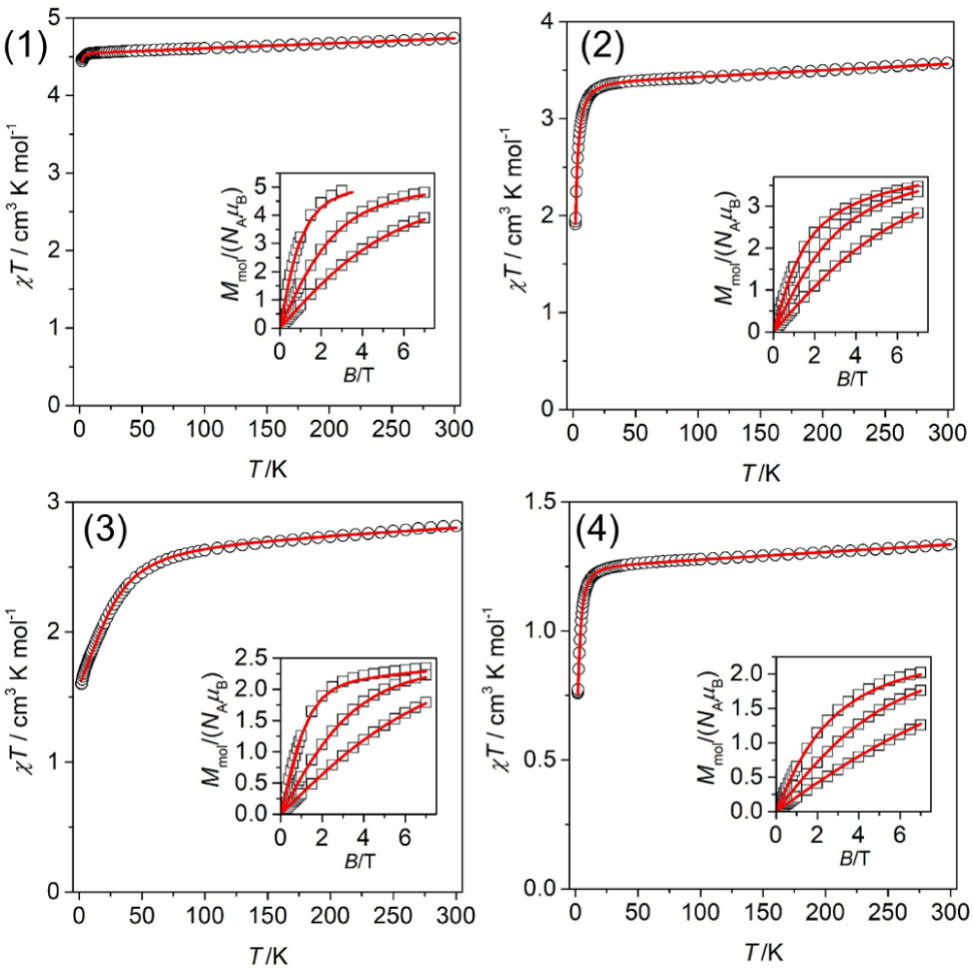
Magnetic data for compounds **1–4**. Temperature dependence of the effective magnetic moment and the isothermal magnetizations measured at *T* = 2, 5, and 10 K, as shown in the inset. The empty symbols represent the experimental data points, and the red lines represent the best fits calculated with the parameters listed in Table 3.

On lowering the temperature, the χ*T* value for **1** is almost constant in the whole temperature range. Also, the isothermal magnetization is close to the Brillouin function, indicating a negligible zero-field splitting (ZFS), as expected for 3d^5^ electronic configuration. On the other hand, a significant drop of the χ_M_ *T* product is observed for all the other compounds **2–4** at low temperatures, which indicates a significant magnetic anisotropy and, hence, a strong ZFS. This is also supported by lower values of the isothermal magnetizations at the highest applied magnetic field in comparison with theoretically expected values derived by the Brillouin function as *M*_mol_/*N*_A_μ_B_ = *g*·*S*, where *g* = 2 and *S* = 2 for **2**, *S* = 3/2 for **3,** and *S* = 1 for **4**.

To extract ZFS parameters from the experimental magnetic data, the spin Hamiltonian describing the magnetic anisotropy was postulated as

1 $$Ĥ=D\left({{Ŝ}_{z}}^{2}-{Ŝ}^{2}/3\right)+E\left({{Ŝ}_{x}}^{2}-{{Ŝ}_{y}}^{2}\right)+\mu_{B}Bg{Ŝ}_{a}-zj\left\langle {Ŝ}_{a}\right\rangle {Ŝ}_{a}$$

where *D* and *E* are the single-ion axial and rhombic ZFS parameters, respectively. The Zeeman term is defined in the direction of the magnetic field as *B*_a_ = *B*(sin­(θ) cos­(φ), sin­(θ) sin­(φ), cos­(θ)) with the help of the polar coordinates, and the last component represents the molecular-field correction quantified by the *zj* parameter.^73^ Then, the molar magnetization of the powder sample was calculated.

To assess the ZFS parameters by simultaneously fitting the χ_M_ *T* = f­(T) and *M* = f­(H) dependencies, the software PHI^46^ was used. The experimental magnetic data were fitted with both signs of the *D*-parameter, but only those results providing more accurate fits (the best-fit parameters) are listed in Table 3. Because of the lack of any extensive system of hydrogen bonds providing any supramolecular structures, the effect of intermolecular magnetic interactions was negligible, as deduced from the zero values of the molecular-field correction term zj (this parameter was finally not applied during the fitting procedure).

The positive *D*-values were obtained for complexes **1**–**3**, but for Mn­(II) complex **1**, the value was close to zero, confirming a very small magnetic anisotropy (ZFS), which was suggested from the temperature dependence of the χ_M_ *T* product. On the other hand, the *D*-value for Fe­(II) complex **2** was relatively moderate, and a large value was obtained for Co­(II) complex **3**. A rather large negative value was obtained for Ni­(II) complex **4**. The signs as well as the values of the *D* parameter are in accordance with the previously obtained findings for seven-coordinate pentagonal bipyramidal complexes. Very small values were found for Mn­(II) complexes,^13^ large positive values were found for Co­(II) complexes,^1,33–35^ large negative values for Ni­(II) complexes,^1,38^ and usually negative values were obtained for Fe­(II) compounds.^1^

In order to obtain a better correlation, the obtained *D*-values were compared with those of previously studied complexes with **L1**–**L3** (Table 3),^39–41^ and they are reported in Figure 5 as a function of coordinated macrocyclic ligands **L1**–**L4**, i.e., as a function of the functional group in the pendant arm.

**Figure 5:**
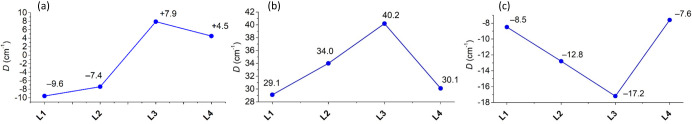
Variation of the fitted parameter *D* in Fe­(II) (a), Co­(II) (b), and Ni­(II) (c) complexes depending on the coordinated macrocyclic ligand **L1**–**L4**.

There is no evident trend regarding the Fe­(II) complexes (Figure 5a); moreover, both negative and positive values have been found, and the situation is complicated by the fact that energetically low-lying excited states are present for Fe­(II) complexes, and thus, the spin Hamiltonian formalism is limited. A more apparent trend can be observed for Co­(II) (Figure 5b) and Ni­(II) (Figure 5c) complexes, where for the order **L1** → **L2** → **L3** → **L4**, i.e., carboxylate → pyridine → benzimidazole → pyridine-*N*-oxide, the *D*-value is increasing from **L1** to **L3** (more positive for Co­(II) compounds and more negative for Ni­(II) compounds), but for **L4,** it drops down to the values very similar to that for **L1**. This trend can be explained by (i) different donor–acceptor properties of the functional group in **L4** and (ii) different structural changes of the coordination geometry.

According to the calculations previously reported by Sutter and Mallah,^19^ which consider the contributions to the *D*-value from available excited states, the stronger axial ligand field should provide larger magnetic anisotropy in the case of Ni­(II) complexes, while an opposite effect is expected for Co­(II) complexes. Regarding the series of Co­(II) and Ni­(II) complexes with **L1**–**L4**, this prediction is valid for the first group of Co­(II) complexes. If the bond length is considered as the sole parameter for the description of the ligand field strength (shorter bond length = stronger bond = stronger ligand field), the trend for Co­(II) complexes depicted in Figure 6a can be very well explained because with increasing bond distance, the ligand field is weaker and thus the magnetic anisotropy is larger, i.e., the *D*-value is increasing with the longer Co–donor atom distance in the ligand order **L1** < **L4** < **L2** < **L3**. If the same methodology would apply for Ni­(II) complexes, the largest *D*-value should be expected for **L1** decreasing to **L3** in the order mentioned above. But it does not follow the experimental values because the Ni­(II) complex with **L1** has the lowest *D*-value. Therefore, in this case, not only the ligand field in the axial positions but also the ligand field in the equatorial plane has to be taken into account. If the *D*-values of Ni­(II) complexes are ordered according to the CSM deviations from the ideal pentagon (see Table 2), the *D*-value increases and followed the experimental order **L4** < **L1** < **L2** < **L3** (Figure 6c). Thus, for Ni­(II) complexes, in which the Jahn–Teller effect provides a strong distortion mainly in the equatorial plane, this distortion of the pentagonal equatorial ligand field has a stronger effect on magnetic anisotropy than the changes of the axial ligand field. Thus, for the Ni­(II) complex, as much symmetrization of the equatorial ligand field as possible is required.

**Table 3:** Comparison of *Ab Initio* Calculated and Fitted Spin Hamiltonian Parameters for Complexes **1–4** Comprising the **L4** Ligand and for the Previously Studied Complex with **L1–L3** ^a^

Complex	1	2	3	4
	Mn(II)	Fe(II)	Co(II)	Ni(II)
Fit of the experimental data				
*D* (cm^–1^)	+0.381(2)	+4.47(1)	+30.10(7)	–7.576(4)
*E*/*D*	–	0.108(1)	0.005(1)	0.110(1)
*g*	2.038(1)	2.120(1)	2.374(1)	2.234(1)
χ_TIP_	0.645(1)	0.643(1)	0.552(5)	0.2899(4)
Theoretical calculations with CASSCF/NEVPT2				
*D* (cm^–1^)	–0.110	–6.13	+28.2	–7.32
*E/D*	0.187	0.265	0.0299	0.0266
*g*_*x*_	2.002	2.070	2.348	2.236
*g*_*y*_	2.002	2.040	2.378	2.233
*g*_*z*_	2.002	2.204	2.102	2.287
*U*_calc._(cm^–1^)	0.71	27.2	56.5	7.51
Experimental data for complexes with **L1** ^39^				
*D* (cm^–1^)	–	–9.6	29.1	–8.5
*E*/*D*	–	0.006	0	0.19
Experimental data for complexes with **L2** ^40^				
*D* (cm^–1^)	0	–7.4	34.0	–12.8
*E*/*D*	0	0.0	0.0	0.136
Experimental data for complexes with **L3** ^41^				
*D* (cm^–1^)	–0.30(3)	7.90	40.3	–17.2
*E*/*D*	0	0.220	0.10	0.076

a The temperature-independent paramagnetism (10^–3^ cm^3^ mol^–1^).

**Figure 6:**
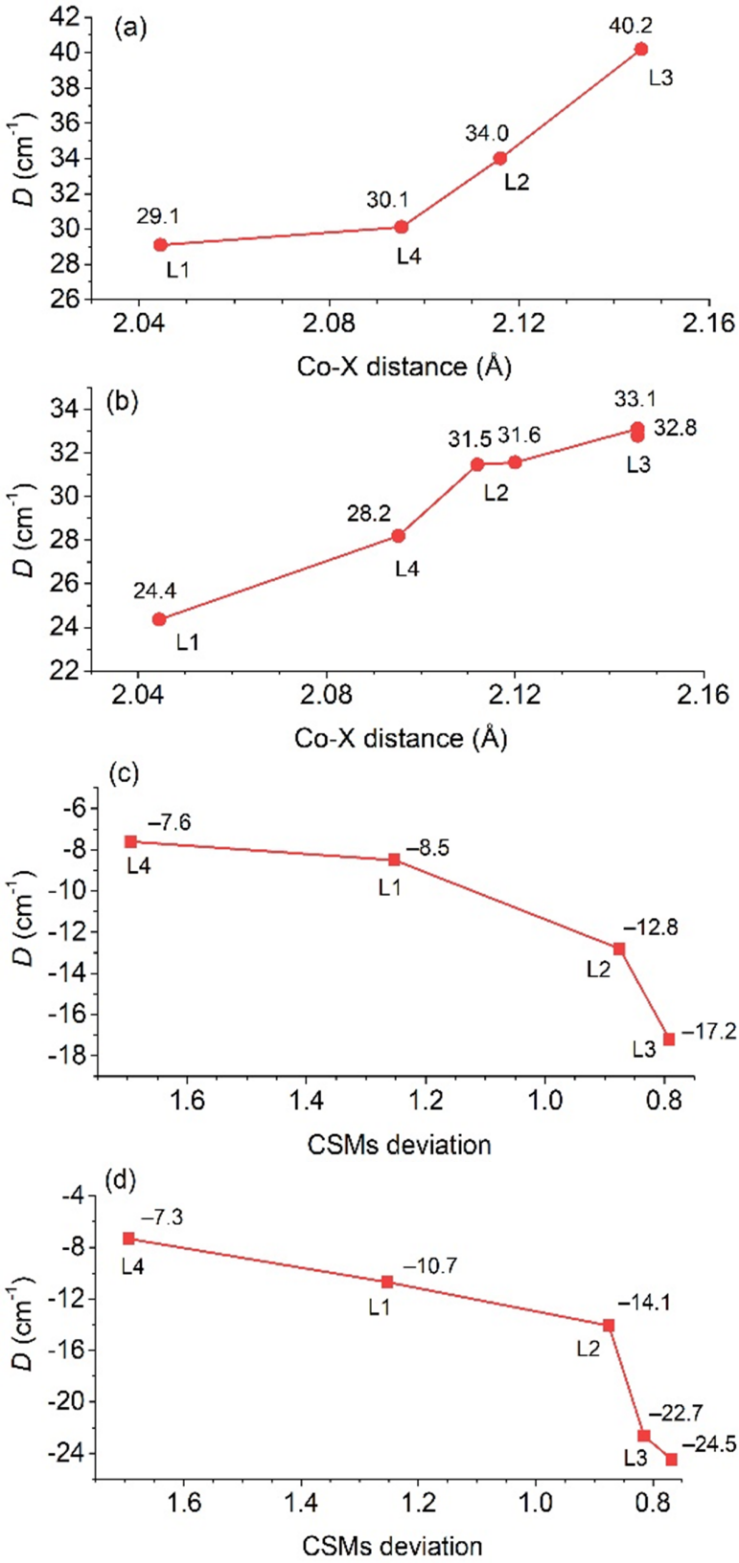
Variation of the *D* parameter in the series of Co­(II) (a, b) and Ni­(II) (c, d) complexes with **L1**–**L4** depending on the Co–X distance (X = donor atom in axial position) or CSM deviations from the ideal pentagon (CN = 5). a) and c) The experimental data, and b) and d) the data obtained by CASSCF/NEVPT2 calculations. In b) and d), two values are given for two crystallographically independent units.

Additionally, comparison with complexes containing **L2** can also be very interesting, providing information about the effect of the transformation of the functional group in the pendant arm from pyridine into pyridine-*N*-oxide. In all Fe­(II), Co­(II), and Ni­(II) complexes, the magnetic anisotropy expressed by the *D*-value is lower for complexes with **L4** in comparison with those with **L2**. Thus, the pyridine functional group, i.e., π-acceptor ligand, is more suitable for providing a larger magnetic anisotropy in comparison with the pyridine-*N*-oxide group, i.e., π-donor ligand.

### Dynamic Magnetic Measurements

During the measurement of alternating current (AC) susceptibility for complexes **2**–**4**, a nonzero out-of-phase signal was found only for Co­(II) compound **3** upon applying a weak static magnetic field (Figure S8). Therefore, temperature- and frequency-dependent AC susceptibility data were acquired at *B*_DC_ = 0.2 T (Figure 7) and clearly defined maxima of the out-of-phase signal of AC susceptibility dependent on the applied frequency were found for compound **3**, which is the characteristic behavior of SMMs. Next, the one-component Debye’s model was applied based on Eqs S1 and S2 and resulted in isothermal (χ_T_) and adiabatic (χ_S_) susceptibilities, relaxation times (τ), and distribution parameters (α) (Table S4). Afterward, the Argand (Cole–Cole) plot was constructed, as shown in Figure 7. The temperature dependence of the extracted relaxation times was fitted by using eq 2, considering the direct and Raman and Orbach relaxation mechanisms. A sufficiently good fit was obtained by a combination of Orbach and direct relaxation mechanisms, giving *U*_eff_ = 26.8(8) K (18.6(6) cm^–1^, Table 4), which is much lower than the expected value based on the magnetic anisotropy (*U*_eff_ = 2*D* = 60.2 cm^–1^). The discrepancy arises from the easy-plane magnetic anisotropy present in compound **3**. In Kramers systems with positive *D* and negligible rhombicity, spin–lattice relaxation often bypasses the high-lying first excited doublet^74^ and instead proceeds via Raman and direct pathways.^20^ Consequently, fitting the data with a combination of direct and Raman mechanisms produced even more accurate and physically reasonable parameters (*DH* ^*m*^ = 145(3) K^–1^s^–1^, *R* = 0.58(7) K^–n^s^–1^, *n* = 5.76(7), Table 4).

$$\tau^{-1}={DH}^{m}T+RT^{n}+\tau_{0}^{-1}\exp\left(-U_{eff}/kT\right)$$ 2

**Figure 7:**
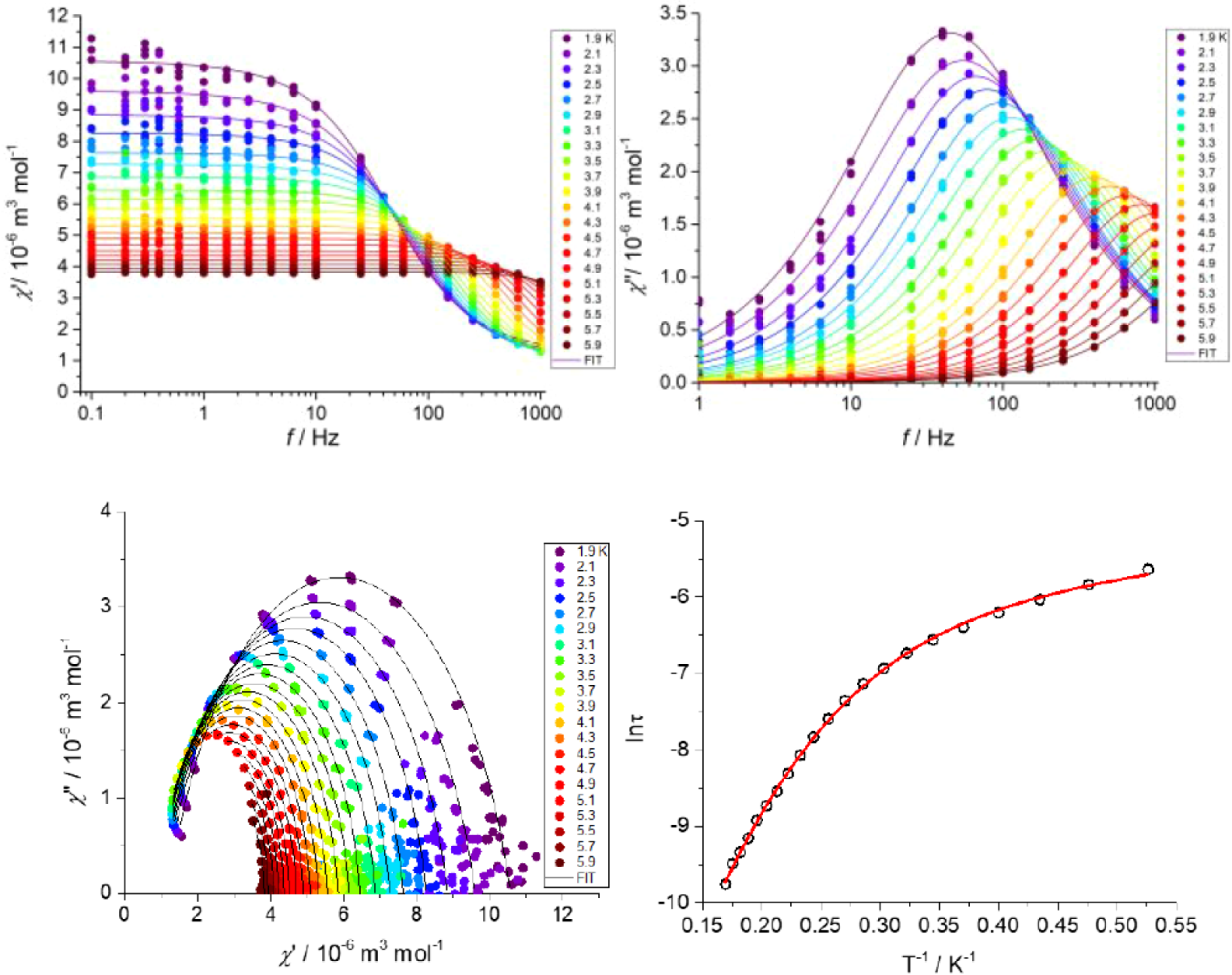
AC magnetic data for complex **3**. Frequency dependence of in-phase χ*’* and out-of-phase χ’’ molar susceptibilities in an external magnetic field of 0.2 T (top). The Argand (Cole–Cole) plot (bottom-left: full lines—fitted data using Debye’s model). A Direct + Raman fit (red line, Table 4) of the resulting relaxation times concerning direct and Raman relaxation processes (bottom-right).

**Table 4:** Parameters for the Relaxation of Magnetization for Complex **3**

Fit	*DH^m^* (K^–1^ s^–1^)	*R* (K^–*n*^ s^–1^), *n*	*U*_eff_/*k*_B_ (K)	*τ*_0_	R^2^
Direct + Orbach	176(6)	–	26.8(8)	7(1) × 10^–7^	0.99697
Direct + Raman	145(3)	0.58(7)	–	–	0.99941
		5.76(7)			

### Multireference Theoretical Calculations

To elucidate the electronic structure of reported complexes **1–4** in more detail, post-Hartree–Fock multireference calculations based on the state-averaged complete active space self-consistent field method (SA-CASSCF) were done with the help of the computational package ORCA. The CASSCF/NEVPT2 calculations provided information about AILFT d-orbitals, ligand-field terms, and ligand-field multiplets, as depicted in Figure 8, and the spin Hamiltonian parameters are listed in Table 3.

**Figure 8:**
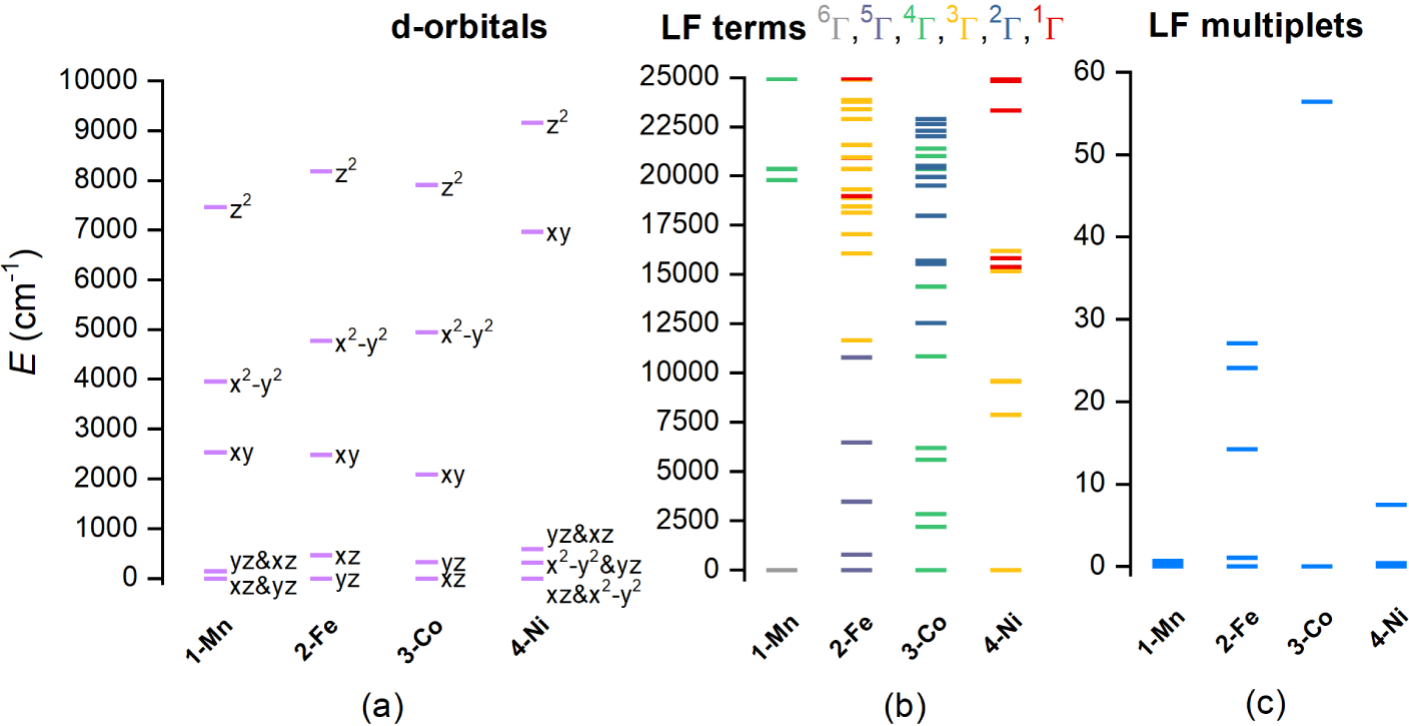
Results of the CASSCF/NEVPT2 calculations for the studied complexes **1–4** showing a) the d-orbital splitting calculated by *ab initio* ligand field theory (AILFT) with labels showing dominant d-orbital contributions (a), low-lying ligand-field terms with various multiplicities (b), and ligand-field multiplets (c).

Detailed analysis of the composition of AILFT d-orbitals and their visualization is reported in Figures S9–S12. It is apparent that the pattern of the d-orbital splitting varies across the series and is far from the ideal splitting of *e*_1_″ (d_*xz*_, d_*yz*_), *e*_2_′ (d_*xy*_, d_*x*2 –_ *_y_*_2_), and *a*_1_′ (d_*z*2_) derived for the *D*_5h_ group. However, for complexes **1**–**3** with the lowest deviation from a pentagonal bipyramidal shape (CMSs, Table 2), splitting is still resembling the expected splitting pattern. The splitting of *e*_2_′ (*d*_*xy*_, *d*_*x*2 –_ *_y_*_2_) orbitals is increasing from **1** to **3**, most likely due to lowering the ligand field symmetry in the plane of the macrocyclic ligand, which is supported by the increasing difference of M–N and M–O1/O2 DI values (Figure 3, bottom). The situation for complex **4** is much more pronounced. The energy splitting of d_*x*2 –_ *_y_*_2_ and d_*xy*_ is the largest. Also, we observe a rotation of the *XY* axis within the plane of the macrocyclic ligand. Thus, the d_*x*2 –_ *_y_*_2_ orbital is oriented toward the N1 atom for **1**–**3**, whereas the d_*xy*_ orbital is oriented toward the N1 atom for **4**. Overall, these findings support lowering the coordination number due to semicoordination/very weak coordination of Ni–O1/O2 in **4**.

The scheme of splitting of the ligand field terms and the ligand field multiplets (Figure 8) is analogous to a similar series with **L1**–**L3** macrocyclic ligands. Calculations revealed significant ZFS parameters for **2–4** (Table 3), which are in good agreement with the experimental values. Calculated individual nonzero contributions to the *D*-tensor are listed in Table S5. For **3** and **4**, it is evident that several excited states make considerable contributions.

Next, we also performed additional CASSCF/NEVPT2 calculations for the entire series of Co­(II) and Ni­(II) complexes with **L1**–**L4** ligands to reveal in detail the impact of the pendant arms on the electronic structure. First, the results for the Co­(II) series are discussed, for which the delocalization indexes were calculated, showing that the strongest bonds are formed to axial donor atoms of the pendant arms (label X_pa_) followed by bonds to nitrogen of pyridine (label N_py_) and to amine-nitrogen atoms (label N_am_) of the macrocycle (Figure 9). The weakest bonds are those to oxygen atoms of the macrocycle (label O_et_), and they are very similar to each other and quite similar also within the Co­(II) series. This is reflected in AILFT d-orbital splitting, which resembles the ideal splitting for *D*_5h_ symmetry. However, the largest deviation within the Co­(II) series is observed for the [Co­(**L1**)] complex, for which the weakest Co–O_et_ bonds were found. Interestingly, the *D*-tensor axes for this complex are tilted; the *z*-axis does not pass through X_pa1_–Co–X_pa2_, as is the case for the rest of the Co­(II) complexes (Figure S13). As the pattern of the excited LFT varies only slightly, the ZFS *D*-parameter is found to be positive and significantly large for the entire series (Figure 6b).

**Figure 9:**
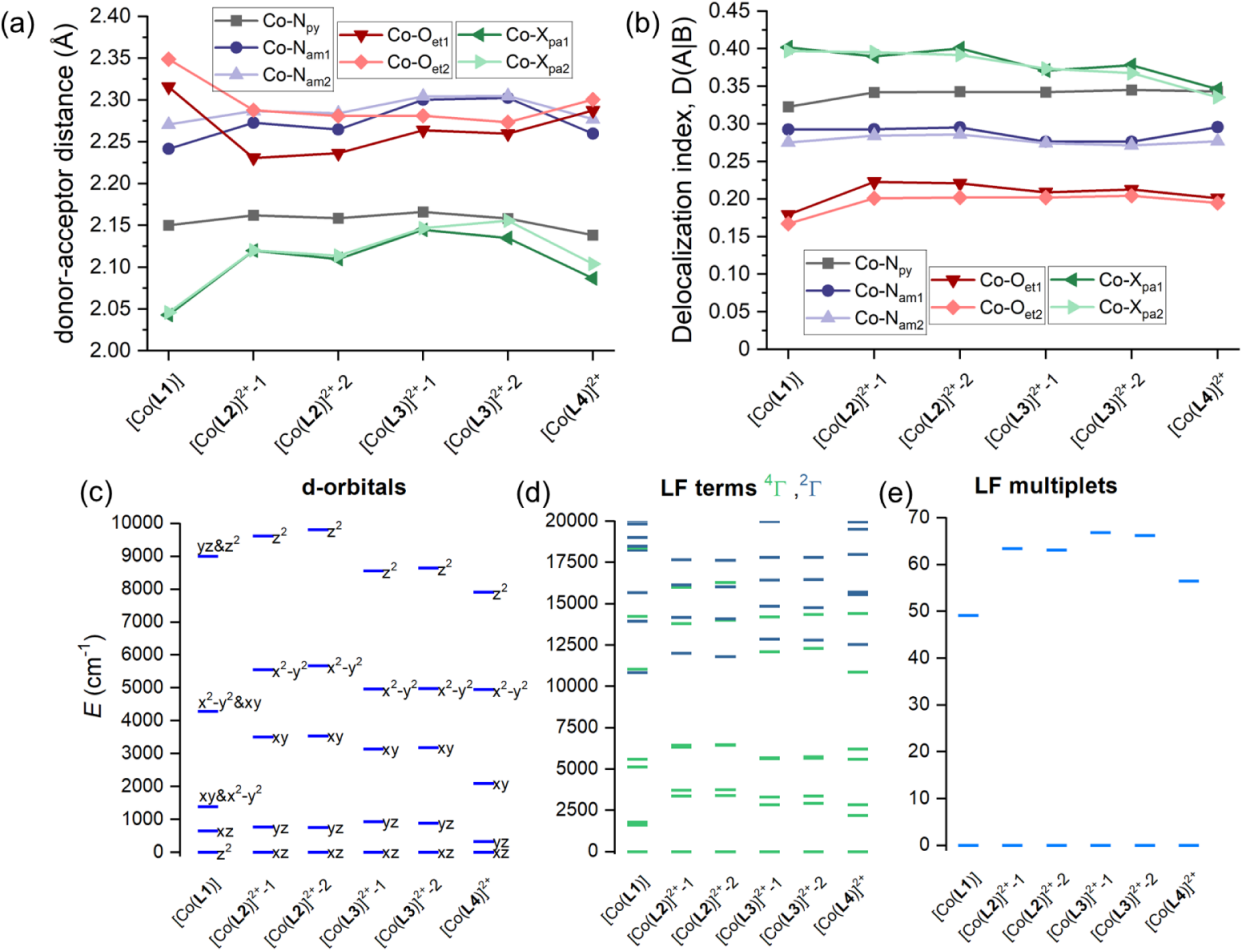
Summary for Co­(II) complexes with **L1**–**L4** ligands. (a) Comparison of the metal–donor atom distances in the studied complexes. (b) Delocalization index calculated from DFT calculations and subsequent QT-AIM analysis. Results of the CASSCF/NEVPT2 calculations for the studied complexes showing the d-orbital splitting calculated by *ab initio* ligand field theory (AILFT) with labels showing dominant d-orbital contributions (c), low-lying ligand-field terms with various multiplicities (d), and ligand-field multiplets (e). Two values for [Co­(**L2**)]^2+^ and [Co­(**L3**)]^2+^ are given for the two crystallographically independent units.

Next, the outcome for the Ni­(II) series is shown in Figure 10. In contrast to the Co­(II) series, the DI values are found to be stronger for Ni–N_py_ bonds than for the axial donor atoms of pendant arms (X_pa_). Additionally, Ni–N_am_ bonds appear to be stronger than their analogous Co–N_am_ bonds. This is reflected in weaker Ni–O_et_ bonds than those for Co–O_et_ bonds. Moreover, it can be seen that there is a larger variation of DI in the Ni­(II) series than in the Co­(II) series of complexes. These structural and bonding differences are also reflected in the splitting of d-orbitals, whose pattern deviates significantly from the ideal splitting in D_5h_ symmetry. Indeed, a larger variation of *D*-parameter is found for the Ni­(II)-series, spanning the interval from −7 to −24 cm^–1^ (Figure 6d), and the *D*-tensor axes are shown in Figure S14. Again, the *z*-axis of the *D*-tensor is significantly tilted from X_pa1_-Ni-X_pa2_ for Ni­(II) complexes with **L1**–**L3** ligands. This means that finding the magneto-structural correlation for such a series is quite problematic.

**Figure 10:**
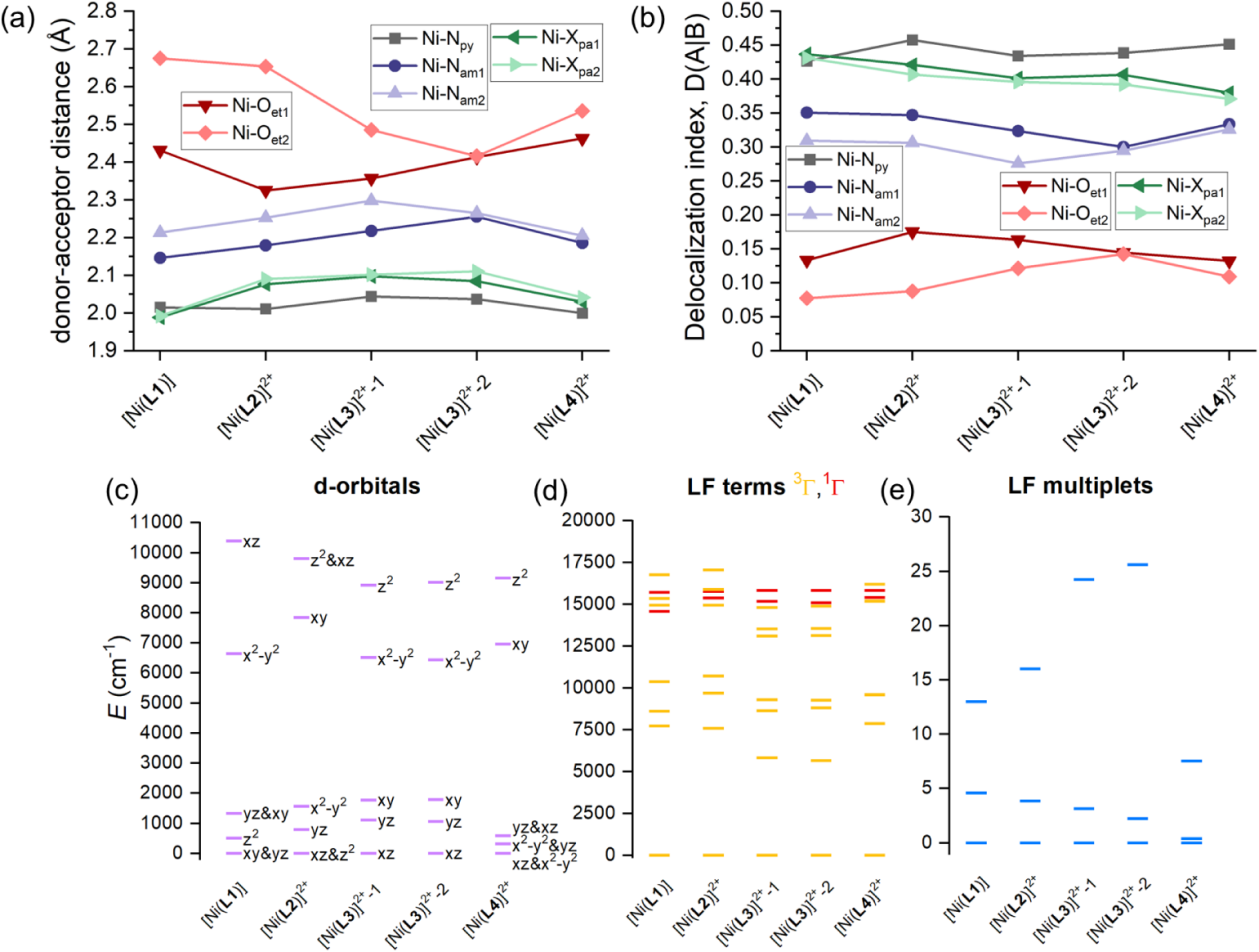
Summary for Ni­(II) complexes with the **L1**–**L4** ligands. (a) Comparison of the metal–donor atom distances in the studied complexes. (b) The delocalization index calculated from DFT calculations and subsequent QT-AIM analysis. Results of the CASSCF/NEVPT2 calculations for the studied complexes showing the d-orbital splitting calculated by *ab initio* ligand field theory (AILFT) with labels showing dominant d-orbital contributions (c), low-lying ligand-field terms with various multiplicities (d), and ligand-field multiplets (e). Two values for [Ni­(**L3**)]^2+^ are given for the two crystallographically independent units.

## Conclusions

A structurally new macrocyclic ligand with two pyridine-*N*-oxide pendant arms (**L4**) has been synthesized together with its Mn­(II), Fe­(II), Co­(II), and Ni­(II) complexes **1**–**4** and thoroughly characterized. The molecular structures of all studied complexes revealed axially compressed pentagonal bipyramidal coordination geometry, with the largest distortion in the equatorial plane observed for Ni­(II) complex **4** due to a strong Jahn–Teller effect, resulting in a coordination number of 5 + 2 (both macrocyclic oxygen donor atoms are very weakly coordinated, approaching semicoordination). The magnetic data analysis revealed small and moderate magnetic anisotropy for Mn­(II) and Fe­(II) complexes **1** and **2**, respectively, and pronounced magnetic anisotropy for Co­(II) and Ni­(II) complexes **3** and **4** (*D* = +30.1 and −7.6 cm^–1^, respectively). Co­(II) complex **3** was found to behave as a field-induced SMM with a preferential direct and Raman mechanism of relaxation of magnetization. The obtained results were supported by theoretical CASSCF/NEVPT2 calculations, which correspond very well to the observed values of magnetic anisotropy. Moreover, comparison with structurally similar systems studied previously revealed interesting magneto-structural correlations, especially for the investigated pentagonal bipyramidal Co­(II) and Ni­(II) complexes. For the first group, modulation of the axial ligand field seems to be more crucial, and the *D*-value increases with a larger distance between the Co atom and the axial donor atom. In the case of Ni­(II) complexes, symmetrization of the pentagonal equatorial ligand field is more important, and the *D*-value increases with a more symmetrical equatorial plane. Moreover, modification of the pyridine pendant arm into pyridine-*N*-oxide resulted in smaller magnetic anisotropy for all of the studied central metal ions. Thus, the strategy for larger magnetic anisotropy in this class of complexes with macrocyclic ligands relies on employing pendant arms containing π-acceptor functional groups rather than π-donor moieties and N-donors rather than O-donors.

## Supplementary Material



## Notes

2D NMR spectra of L4, IR spectra, PXRD patterns, additional molecular structures using ellipsoids, compositions, and visualization of AILFT d-orbitals, visualization of *D*-tensors, crystal data and structure refinements, results of QT-AIM analysis, SHAPE calculations, parameters of extended one-set Debye model, individual nonzero contributions to *D*-tensors (PDF)
