# Supplementary material for: Pentagonal Bipyramidal First-Row Transition Metal Complexes with Macrocyclic Ligand Containing Two Pyridine‑N‑Oxide Pendant Arms: Structural, Magnetic, and Theoretical Studies
Source: Inorg Chem. 2025 Nov 10;64(46):22683–97. doi: 10.1021/acs.inorgchem.5c03514 (PMC12648663; doi:10.1021/acs.inorgchem.5c03514)
Supplement: Supplementary file 1 [file ic5c03514_si_001.pdf]

Supporting Information for

Pentagonal bipyramidal first-row transition metal complexes with macrocyclic ligand containing two pyridine-*N*-oxide pendant arms: structural, magnetic, and theoretical studies

Bohuslav Drahoš[a]* Ivan Šalitroš[b] and Radovan Herchel[a]

[a] *Department of Inorganic Chemistry, Faculty of Science, Palacký University Olomouc, 17. listopadu 12, CZ-771 46 Olomouc, Czech Republic, Fax: +420 585 634 954. Tel: +420 585 634 429. E-mail: bohuslav.drahos@upol.cz*

[b] *Department of Inorganic Chemistry, Faculty of Chemical and Food Technology, Slovak University of Technology in Bratislava, Bratislava SK-812 37, Slovakia.*

Table of content:

Figure S1 ^1H–^1H *gs*-COSY NMR spectrum of **L4**.

Figure S2 ^1H–^{13}C *gs*-HMQC NMR spectrum of **L4**.

Figure S3 ^1H–^{13}C *gs*-HMBC NMR spectrum of **L4**.

Figure S4 Comparison of IR spectra of the studied complexes **1**–**4**.

Figure S5 The X-ray powder diffraction patterns for complexes **1**–**4**.

Figure S6 The molecular structures of the $[M\mathbf{L4}]^+$ cations found in the crystal structure of complex **1**–**4**.

Figure S7 The molecular structures of the asymmetric units of complexes **1**–**4**.

Figure S8 Out-of-phase component of AC susceptibility measured under various B_{DC} fields at 2 K for compounds **2**–**4**.

Figure S9 The composition of AILFT d-orbitals and their respective energies according to CASSCF/NEVPT2 calculations, and the visualization of the respective orbitals together with XYZ axis of D-tensors for **1-Mn**.

Figure S10 The composition of AILFT d-orbitals and their respective energies according to CASSCF/NEVPT2 calculations, and the visualization of the respective orbitals together with XYZ axis of D-tensors for **2-Fe**.

Figure S11 The composition of AILFT d-orbitals and their respective energies according to CASSCF/NEVPT2 calculations, and the visualization of the respective orbitals together with XYZ axis of D-tensors for **3-Co**.

Figure S12 The composition of AILFT d-orbitals and their respective energies according to CASSCF/NEVPT2 calculations, and the visualization of the respective orbitals together with XYZ axis of D-tensors for **4-Ni**.

Figure S13 The visualization of XYZ axes of D-tensors according to CASSCF/NEVPT2 calculations for Co(II) series of complexes with **L1**-**L3** ligands. Hydrogen atoms are omitted for clarity.

Figure S14 The visualization of XYZ axes of D-tensors according to CASSCF/NEVPT2 calculations for Ni(II) series of complexes with **L1**-**L3** ligands.

Table S1 Crystal data and structure refinements for studied complexes **1**–**4**.

Table S2 The results of QT-AIM analysis for **1**–**4**.

Table S3 Continuous shape measurement for complexes **1**–**4**.

Table S4 Parameters of the extended one-set Debye model for **3** measured at 0.2 T.

Table S5 Calculated individual non-zero contributions to *D*-tensor for studied complexes **1**–**4** obtained from the CASSCF/NEVPT2 calculations.

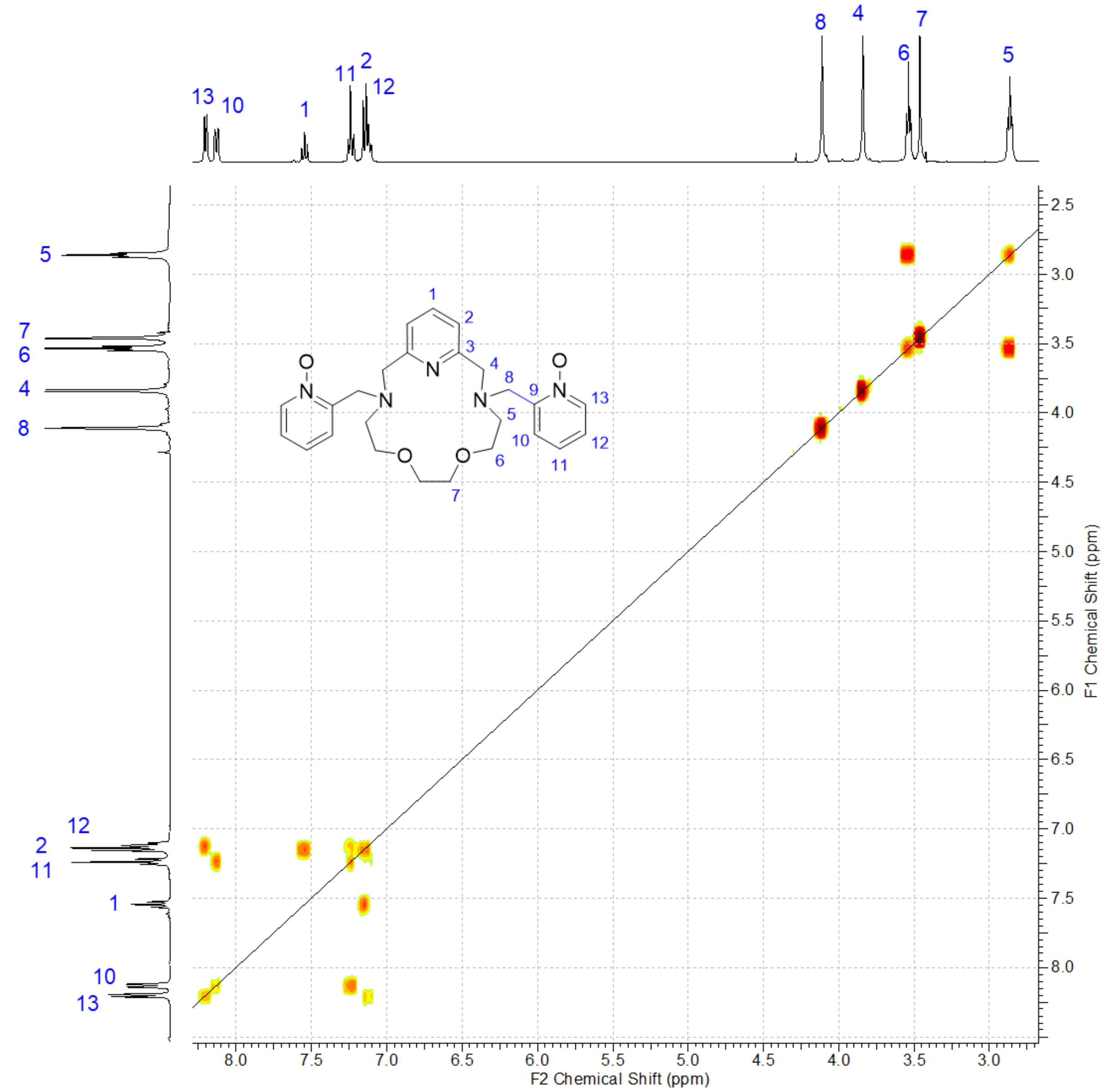

Figure S1: ^{1}H–^{1}H gs-COSY NMR spectrum (400 MHz, $CDCl_3$) of **L4** (3,12-bis((pyridine-1-oxide-2-yl)methyl)-6,9-dioxa-3,12,18-triazabicyclo[12.3.1]octadeca-1(18),14,16-triene) with a residual peak of $CHCl_3$ at 7.27 ppm.

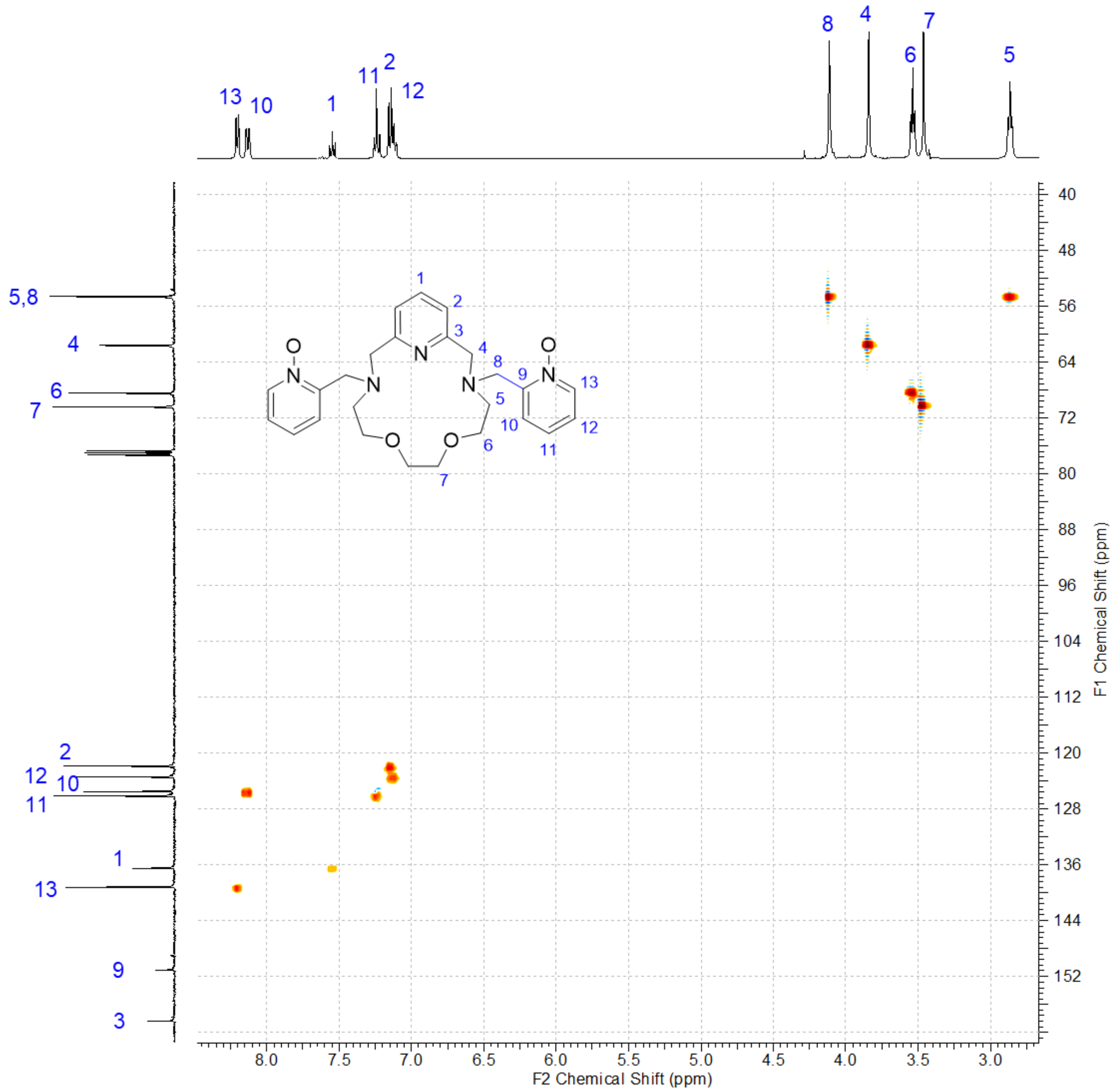

Figure S2: ^{1}H–^{13}C *gs*-HMQC NMR spectrum (400 MHz, $CDCl_3$) of **L4** (3,12-bis((pyridine-1-oxide-2-yl)methyl)-6,9-dioxa-3,12,18-triazabicyclo[12.3.1]octadeca-1(18),14,16-triene) with a residual peak of $CHCl_3$ at 7.27 ppm (^{1}H) and $CDCl_3$ at 77.0 ppm (^{13}C).

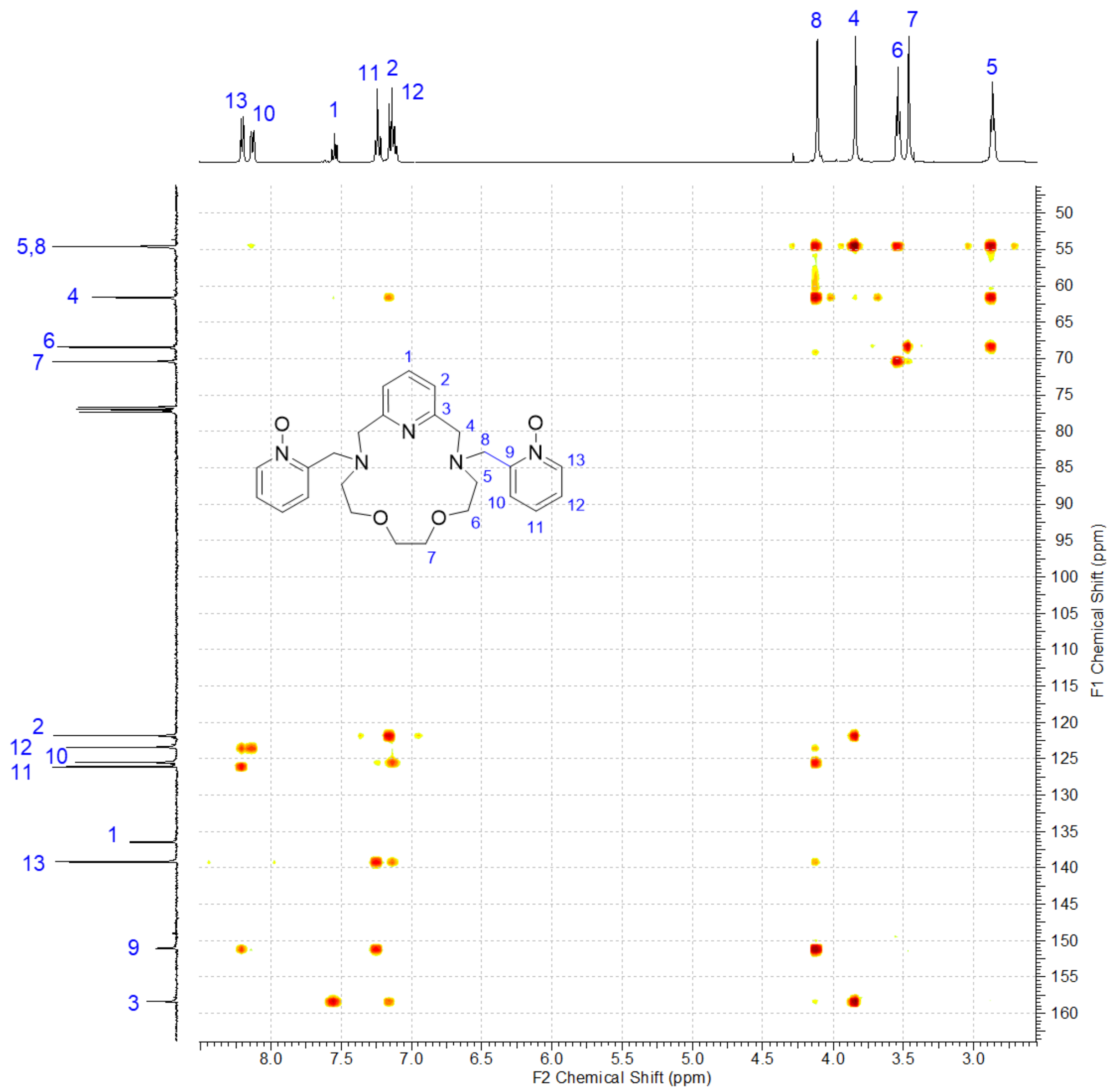

Figure S3: ^{1}H–^{13}C *gs*-HMBC NMR spectrum (400 MHz, $CDCl_3$) of **L4** (3,12-bis((pyridine-1-oxide-2-yl)methyl)-6,9-dioxa-3,12,18-triazabicyclo[12.3.1]octadeca-1(18),14,16-triene) with a residual peak of $CHCl_3$ at 7.27 ppm (^{1}H) and $CDCl_3$ at 77.0 ppm (^{13}C).

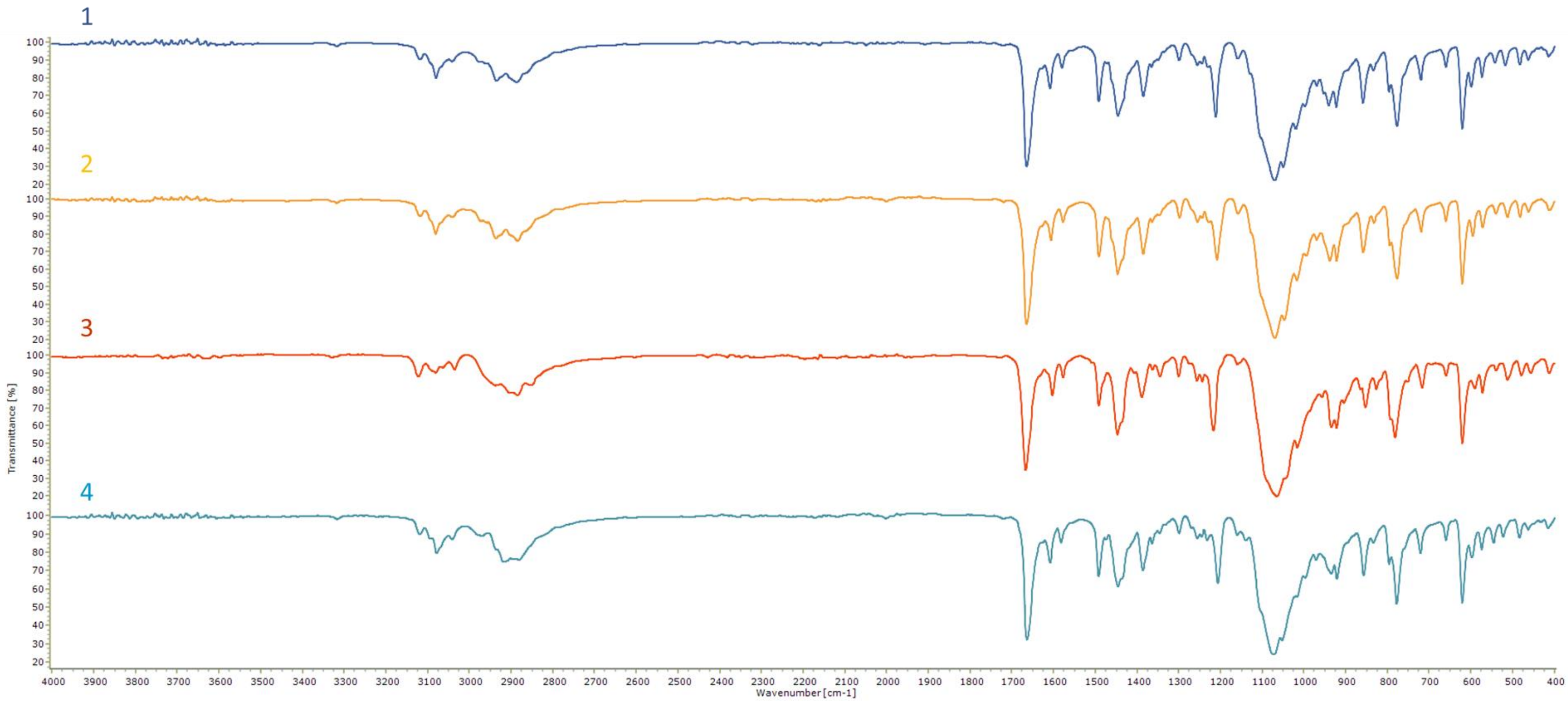

Figure S4 Comparison of IR spectra of studied complexes **1**–**4**. Curve colours: blue = **1**; yellow = **2**; red = **3**; light blue = **4**.

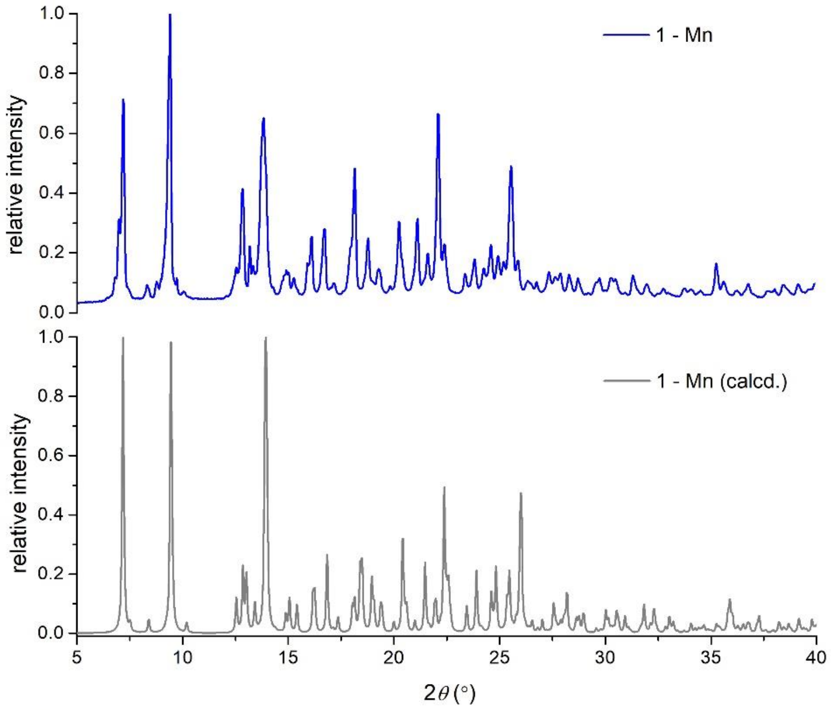
1 - Mn
1 - Mn (calcd.)
relative intensity
2θ (°)

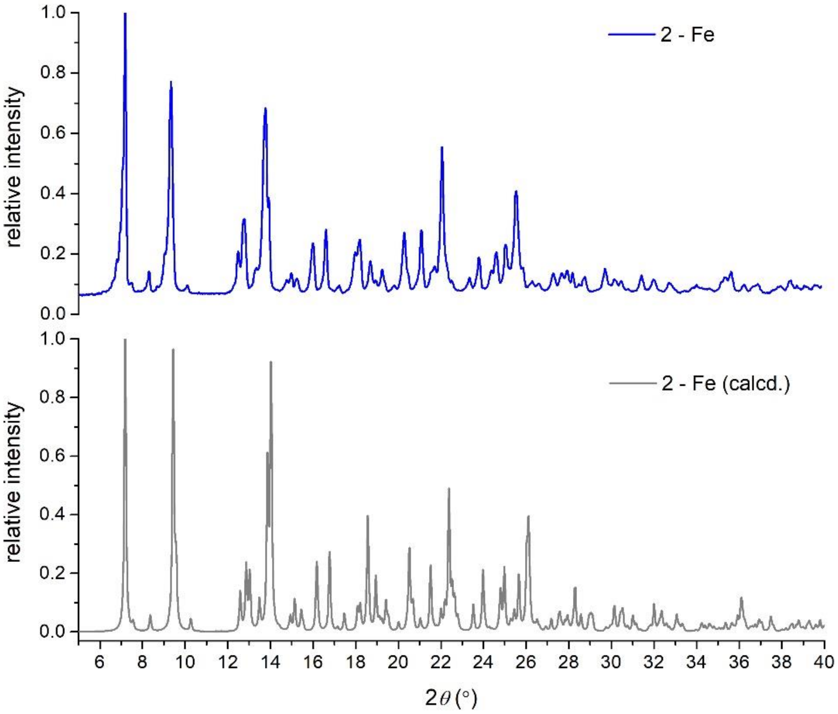
2 - Fe
2 - Fe (calcd.)
relative intensity
2θ (°)

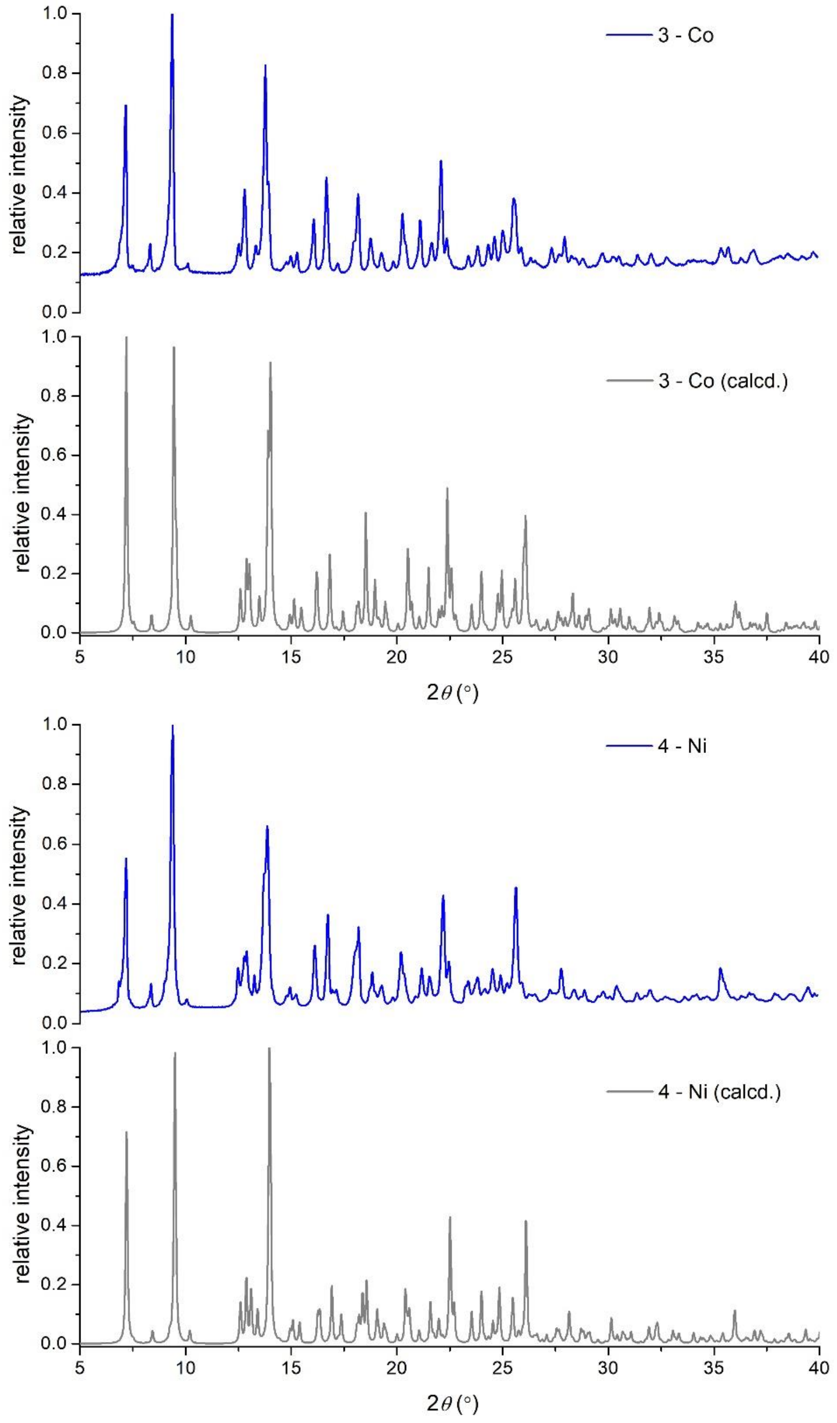

Figure S5 The X-ray powder diffraction patterns for complexes **1**–**4** compared to those calculated from single-crystal X-ray analysis.

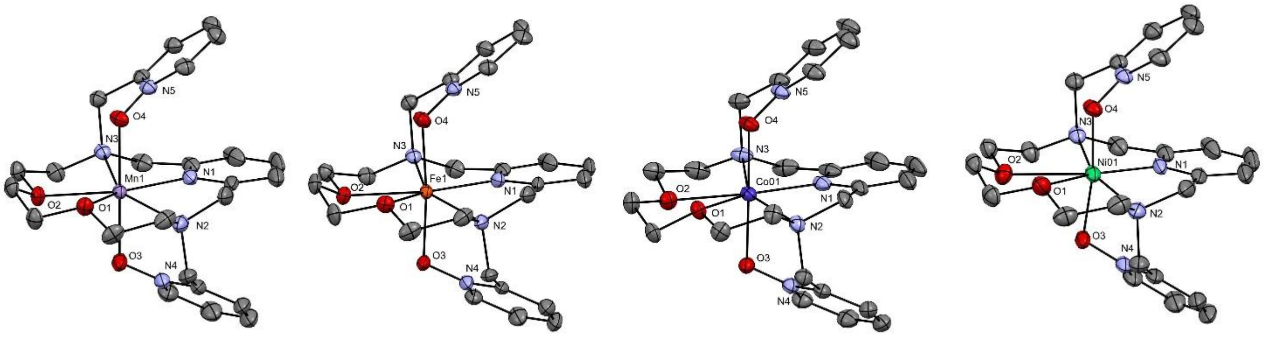

Figure S6 The molecular structures of the [M**L4**]$^+$ cations found in the crystal structure of complex **1** (Mn), **2** (Fe), **3** (Co) and **4** (Ni). Non-hydrogen atoms are drawn as thermal ellipsoids at the 50% probability level. Hydrogen atoms, anions and DMF solvate molecule were omitted for clarity.

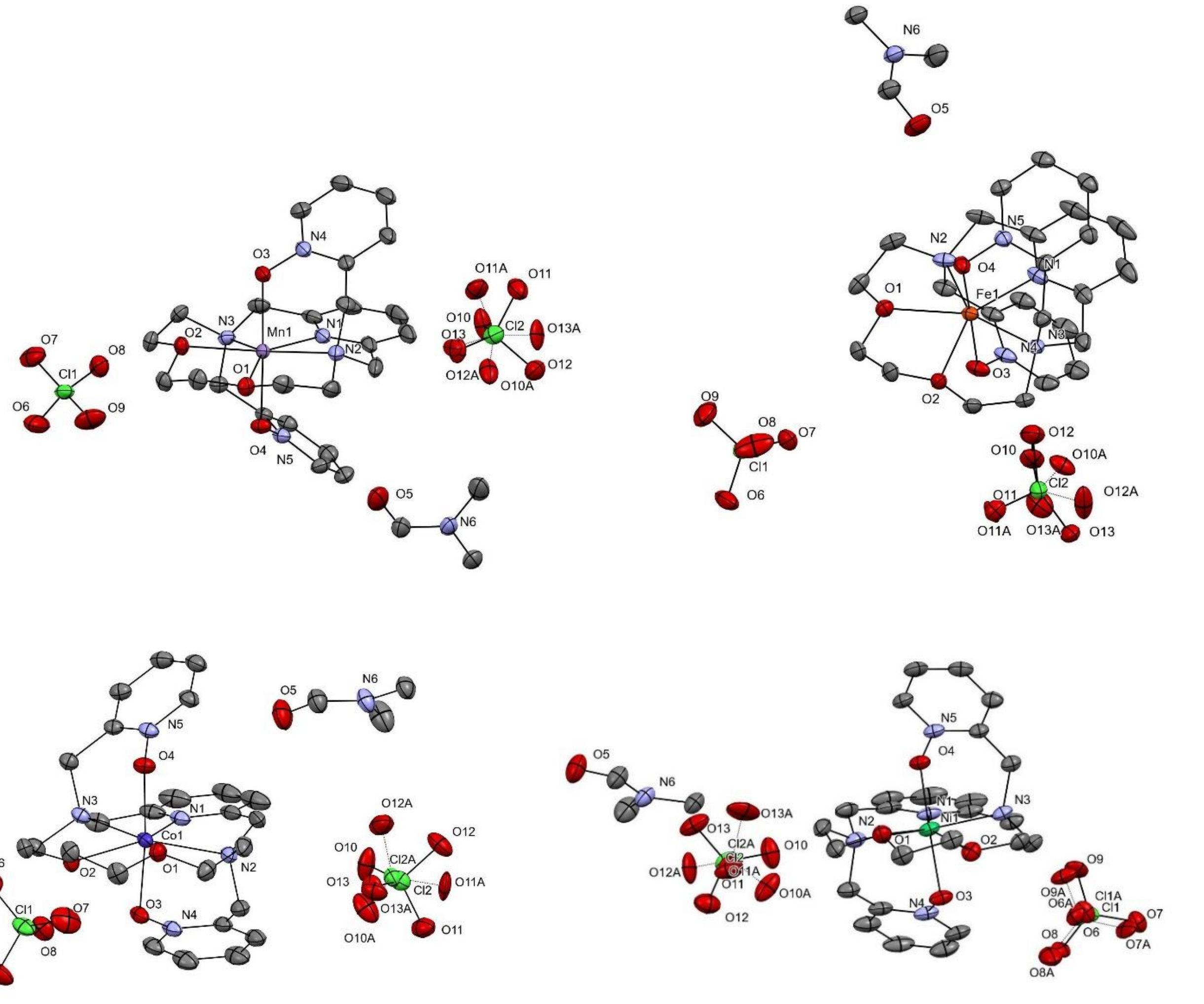

Figure S7 The molecular structures of the asymmetric units of complexes **1**– **4** (from *left* to *right* and *top* to *bottom*). Atoms are drawn as thermal ellipsoids at the 50% probability level. Hydrogen atoms were omitted for clarity.

AC susceptibility measurements

Magnetic measurements under an alternating-current (AC) magnetic field were performed at an amplitude of B_{AC} = 0.1 mT at 2 K. Compounds **2**–**4** were investigated over a static field B_{DC} range of 0–1 T (Fig. S8). At zero static field, none of the compounds exhibited signs of slow relaxation of magnetization. Moreover, compounds **2** and **4** showed no slow relaxation even under an applied static field. In contrast, the application of a static magnetic field induced a clear out-of-phase AC susceptibility signal in compound **3**, indicating field-induced slow magnetic relaxation.

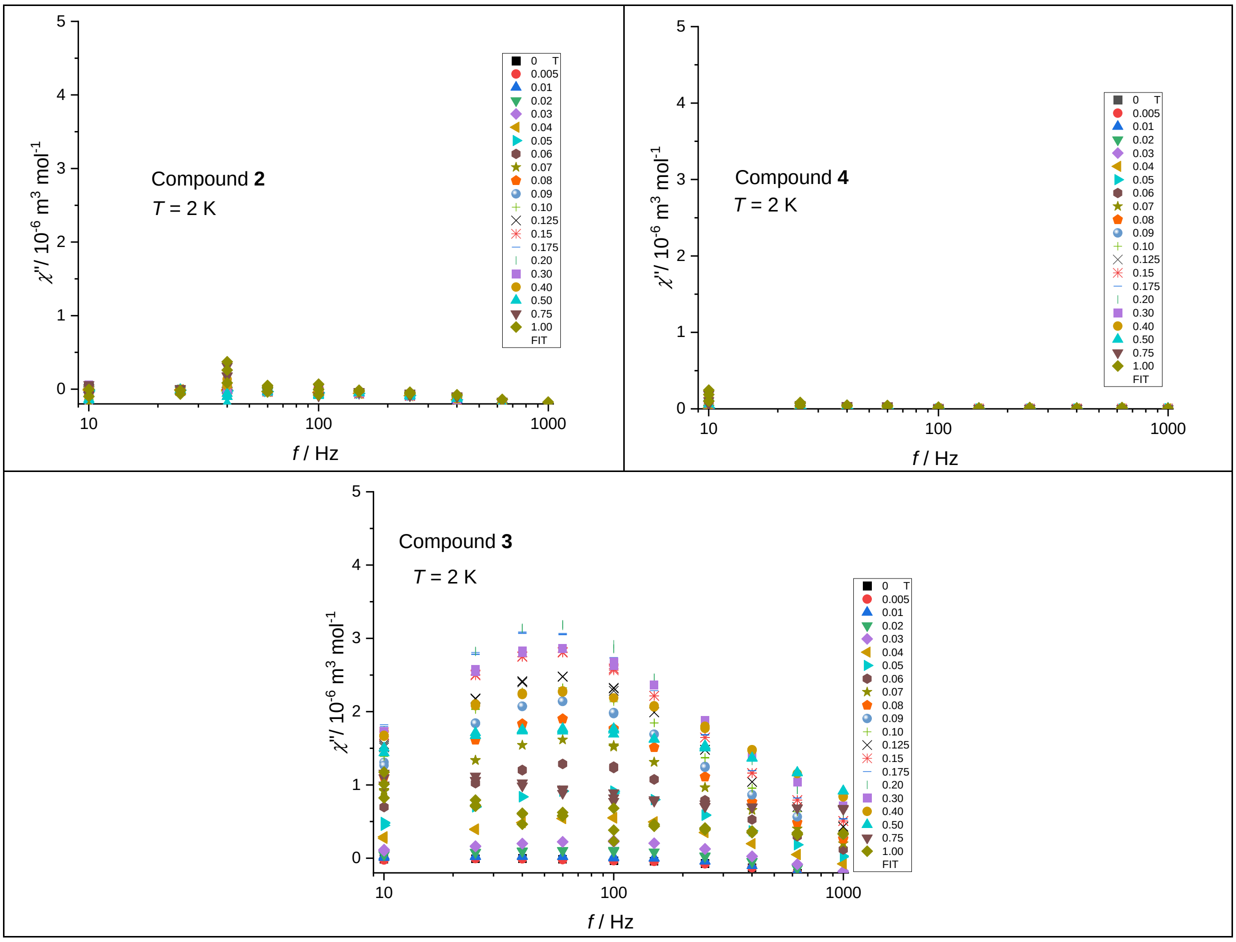

Figure S8 Out-of-phase component of AC susceptibility measured under various B_{DC} fields at 2 K for compounds **2**–**4**.

Further temperature-dependent dynamic magnetic investigations of **3** were performed under a static field B_{DC}=0.2 T across the temperature range of 1.9–5.9 . The collected datasets of χ' and χ'' at each temperature were fitted using the equations for the extended one-set Debye model (Eqs. S1 and S2) and the fitted parameters are listed in Table S4.

$$\chi'(\omega)=\chi_S+\left(\chi_T-\chi_S\right)\frac{1+(\omega\tau)^{(1-\alpha)}\sin(\pi\alpha/2)}{1+2(\omega\tau)^{(1-\alpha)}\sin(\pi\alpha/2)+(\omega\tau)^{(2-2\alpha)}} \qquad \textbf{(S1)}$$

$$\chi''(\omega)=\left(\chi_T-\chi_S\right)\frac{(\omega\tau)^{(1-\alpha)}\cos(\pi\alpha/2)}{1+2(\omega\tau)^{(1-\alpha)}\sin(\pi\alpha/2)+(\omega\tau)^{(2-2\alpha)}} \qquad \textbf{(S2)}$$

Energy (cm^{-1})	d_{z2}	d_{xz}	d_{yz}	d_{x2-y2}	d_{xy}
0	0.02	0.55	0.40	0.02	0.01
139	0.00	0.43	0.50	0.03	0.04
2537	0.00	0.01	0.02	0.09	0.89
3955	0.01	0.00	0.08	0.85	0.07
7465	0.97	0.01	0.01	0.01	0.00

<table>
<tr><th>Energy (cm^{-1})</th><th>AILFT d-orbitals</th><th>Energy (cm^{-1})</th><th>AILFT d-orbitals</th></tr>
<tr><td>0</td><td>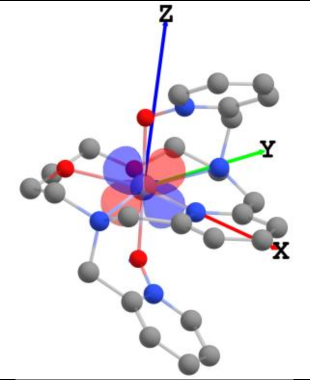
</td><td>3955</td><td>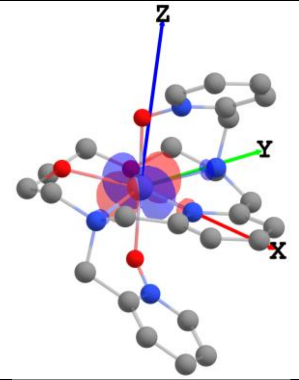
</td></tr>
<tr><td>139</td><td>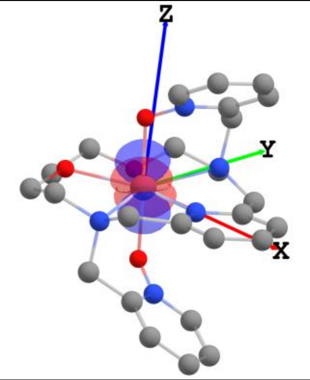
</td><td>7465</td><td>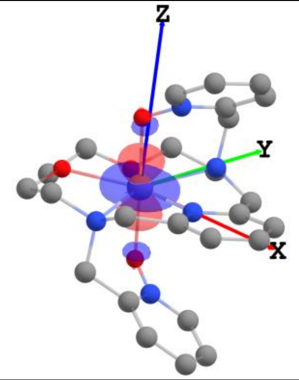
</td></tr>
<tr><td>2537</td><td>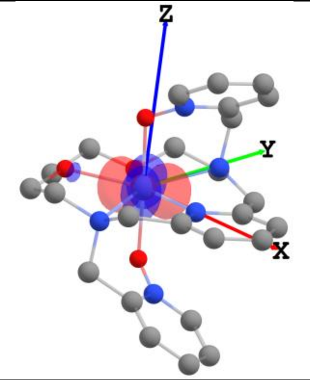
</td><td></td><td></td></tr>
</table>

Figure S9 The composition of AILFT d-orbitals and their respective energies according to CASSCF/NEVPT2 calculations, and the visualization of the respective orbitals together with XYZ axis of D-tensors for **1-Mn**. Hydrogen atoms are omitted for clarity.

Energy (cm^{-1})	d_{z2}	d_{xz}	d_{yz}	d_{x2-y2}	d_{xy}
0	0.02	0.01	0.91	0.00	0.07
465	0.06	0.86	0.03	0.01	0.04
2486	0.00	0.06	0.04	0.23	0.66
4772	0.01	0.00	0.01	0.75	0.23
8183	0.91	0.08	0.01	0.00	0.00

Energy (cm^{-1})	AILFT d-orbitals	Energy (cm^{-1})	AILFT d-orbitals
0	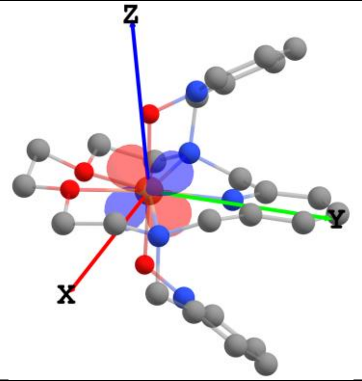	4772	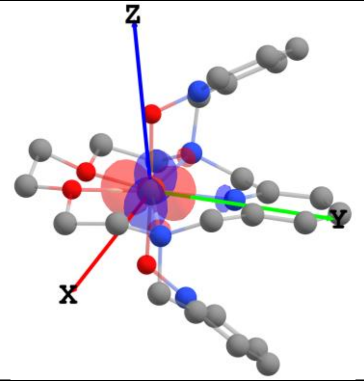
465	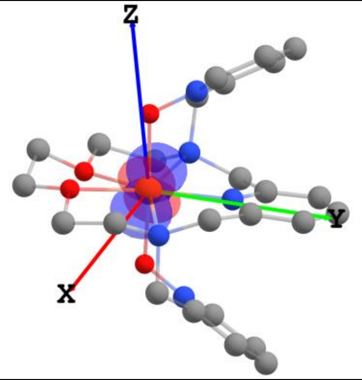	8183	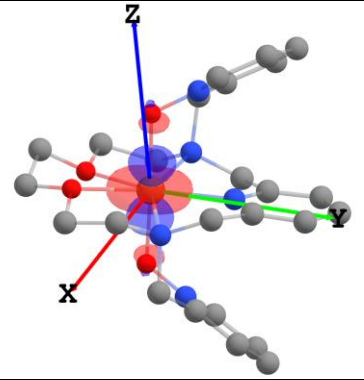
2486	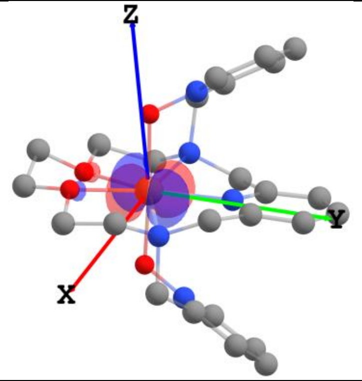		

Figure S10 The composition of AILFT d-orbitals and their respective energies according to CASSCF/NEVPT2 calculations, and the visualization of the respective orbitals together with XYZ axis of D-tensors for **2-Fe**. Hydrogen atoms are omitted for clarity.

Energy (cm^{-1})	d_{z2}	d_{xz}	d_{yz}	d_{x2-y2}	d_{xy}
0	0.00	0.76	0.06	0.04	0.13
328	0.17	0.03	0.76	0.00	0.04
2091	0.00	0.19	0.00	0.14	0.67
4941	0.00	0.00	0.02	0.81	0.16
7910	0.82	0.02	0.15	0.01	0.00

Energy (cm^{-1})	AILFT d-orbitals	Energy (cm^{-1})	AILFT d-orbitals
0	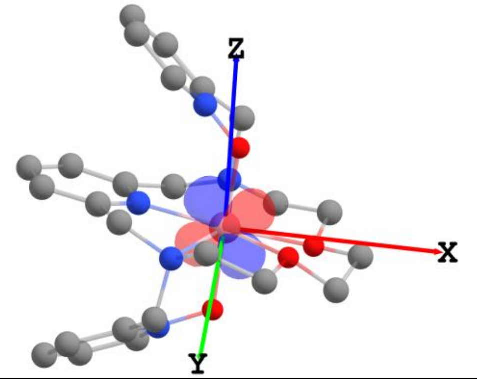	4941	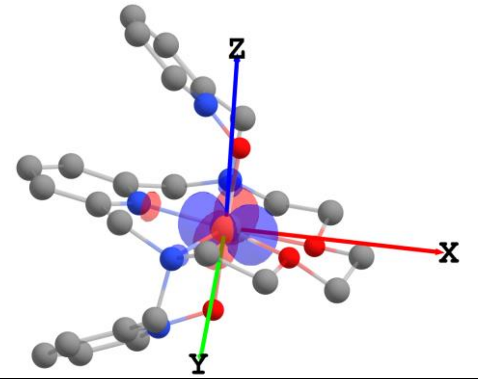
328	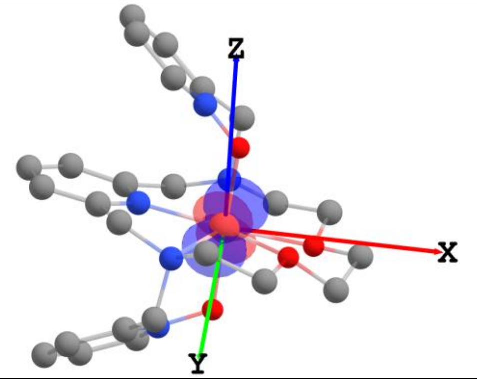	7910	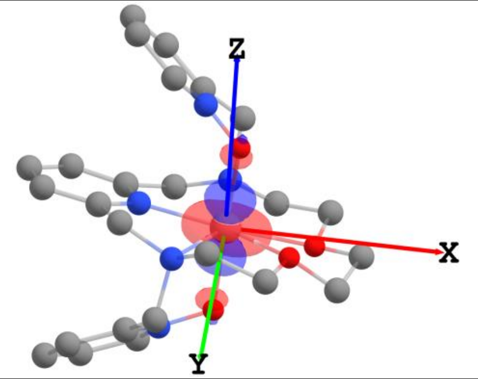
2091	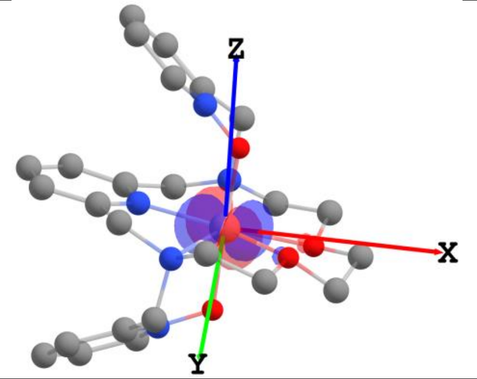		

Figure S11 The composition of AILFT d-orbitals and their respective energies according to CASSCF/NEVPT2 calculations, and the visualization of the respective orbitals together with XYZ axis of D-tensors for **3-Co**. Hydrogen atoms are omitted for clarity.

Energy (cm^{-1})	d_{z2}	d_{xz}	d_{yz}	d_{x2-y2}	d_{xy}
0	0.02	0.56	0.13	0.26	0.04
309	0.01	0.05	0.38	0.53	0.02
589	0.00	0.38	0.48	0.13	0.01
6966	0.01	0.00	0.01	0.07	0.91
9153	0.96	0.01	0.01	0.00	0.01

Energy (cm^{-1})	AILFT d-orbitals	Energy (cm^{-1})	AILFT d-orbitals
0	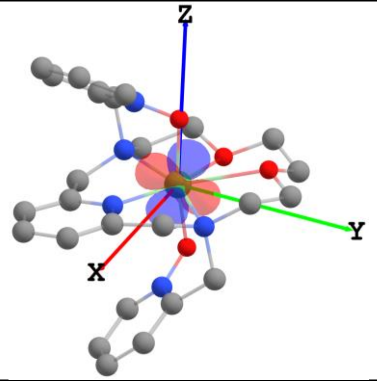	6966	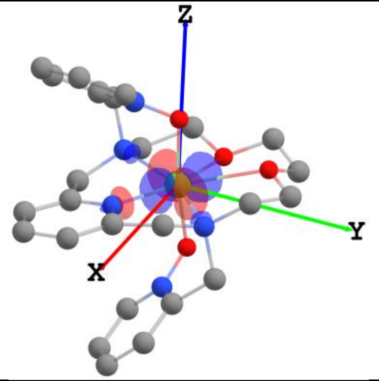
309	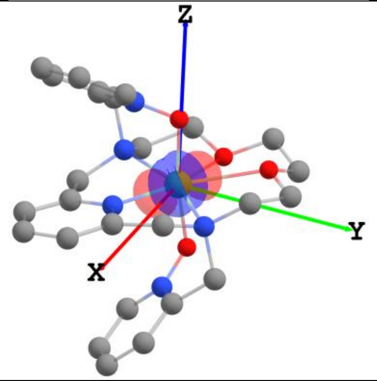	9153	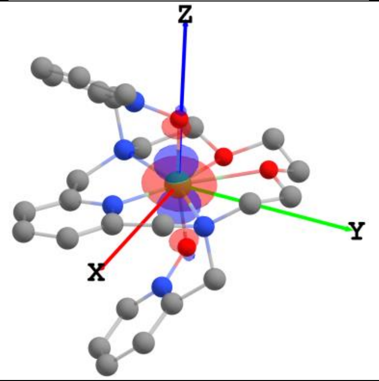
589	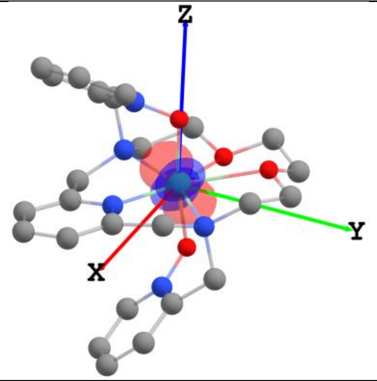		

Figure S12 The composition of AILFT d-orbitals and their respective energies according to CASSCF/NEVPT2 calculations, and the visualization of the respective orbitals together with XYZ axis of D-tensors for **4-Ni**. Hydrogen atoms are omitted for clarity.

[Co(L1)]

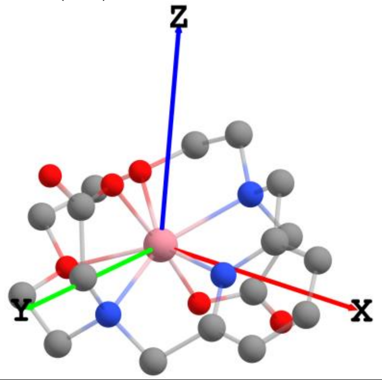

$[Co(L2)]^{2+}$ (for Co1 atom)

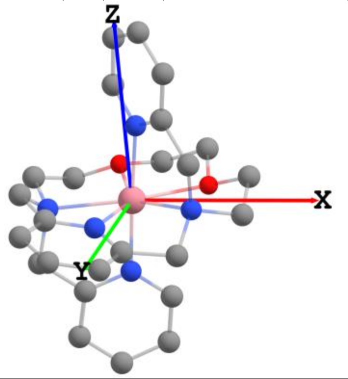

$[Co(L2)]^{2+}$ (for Co2 atom)

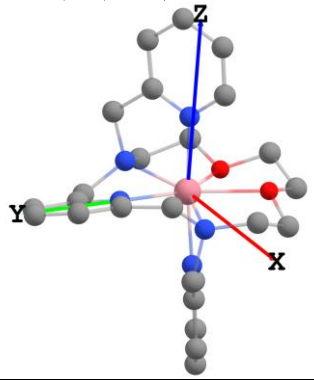

$[Co(L3)]^{2+}$ (for Co1 atom)

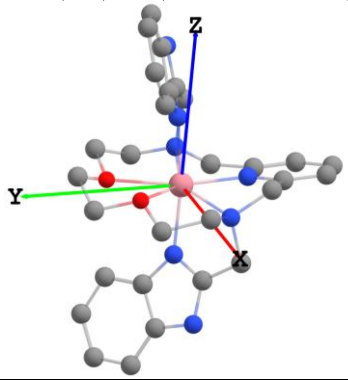

$[Co(L3)]^{2+}$ (for Co2 atom)

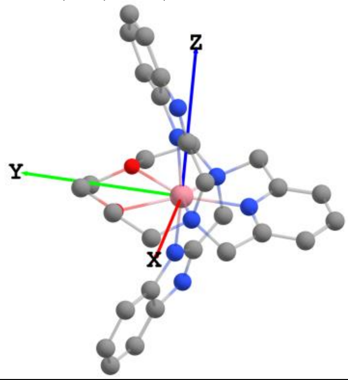

Figure S13 The visualization of XYZ axes of D-tensors according to CASSCF/NEVPT2 calculations for Co(II) series of complexes with **L1-L3** ligands. Hydrogen atoms are omitted for clarity.

<table>
<tr><td>

[Ni(L1)]

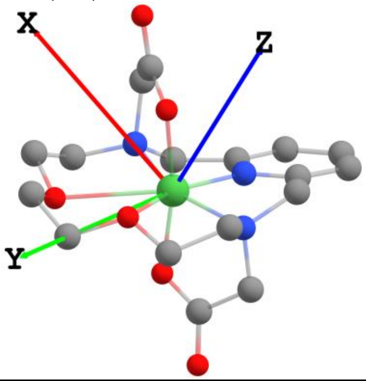

</td><td></td></tr>
<tr><td>

[Ni(L2)]$^{2+}$

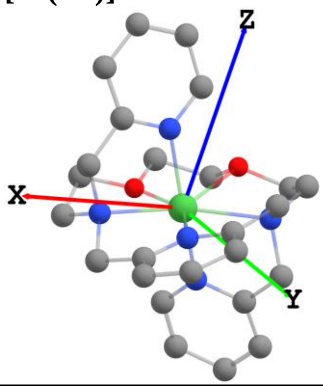

</td><td></td></tr>
<tr><td>

[Ni(L3)]$^{2+}$ (for Ni1 atom)

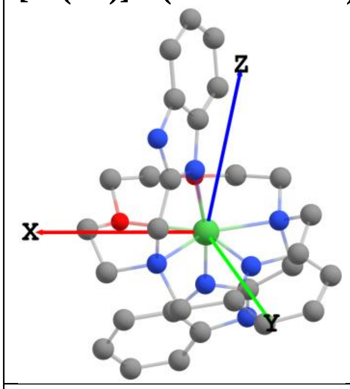

</td><td>

[Ni(L3)]$^{2+}$ (for Ni2 atom)

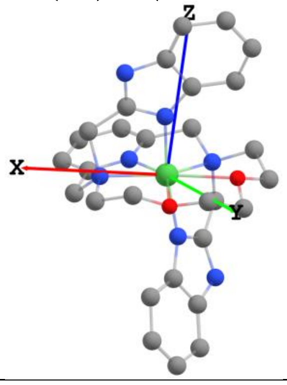

</td></tr>
<tr><td></td><td></td></tr>
</table>

Figure S14 The visualization of XYZ axes of D-tensors according to CASSCF/NEVPT2 calculations for Ni(II) series of complexes with **L1-L3** ligands. Hydrogen atoms are omitted for clarity.

Table S1 Crystal data and structure refinements for studied complexes **1–4**.

Compound	**1**	**2**	**3**	**4**
Formula	$C_{28}H_{38}Cl_2MnN_6O_{13}$	$C_{28}H_{38}Cl_2FeN_6O_{13}$	$C_{28}H_{38}Cl_2CoN_6O_{13}$	$C_{28}H_{38}Cl_2N_6NiO_{13}$
M_r	792.48	793.39	796.47	796.25
Temperature (K)	100.00(10)	100.00(10)	100.00(10)	99.99(10)
Wavelength (Å)	1.54184	1.54184	1.54184	1.54184
Crystal system	monoclinic	monoclinic	monoclinic	monoclinic
Space group	C2/c	I2/a	C2/c	I2/a
a (Å)	23.2127(3)	20.9659(17)	23.1498(3)	23.7459(8)
b (Å)	15.16570(10)	15.1147(2)	15.1103(2)	15.1091(4)
c (Å)	21.1506(3)	37.140(3)	21.0294(3)	21.1591(8)
α (°)	90	90	90	90
β (°)	114.9680(10)	145.329(19)	114.523(2)	118.086(5)
γ (°)	90	90	90	90
V, Å^3	6749.93(15)	6695(2)	6692.53(18)	6697.5(5)
Z	8	8	8	8
D_{calc}, g cm^{-3}	1.560	1.574	1.581	1.579
μ, mm^{-1}	5.287	5.737	6.146	2.959
F(000)	3288.0	3296.0	3304.0	3312.0
2θ range for data collection (°)	7.184 to 136.83	7.192 to 136.408	7.2 to 136.58	7.214 to 136.436
Refl. collected	19842	17109	20738	18620
Independent refl.	6135	6056	6079	6074
R(int)^a	0.0290	0.0343	0.0294	0.0420
Data/restrains/parameters	6135/4/490	6056/4/490	6079/62/493	6074/80/545
Completeness to θ (%)	98.9	98.9	98.9	99.0
Goodness-of-fit on F^2	1.046	1.066	1.033	1.057
R_1, wR_2 (I>2σ(I))^b	0.0311, 0.0783	0.0388, 0.0961	0.0338, 0.0841	0.0433, 0.1103
R_1, wR_2 (all data)^b	0.0326, 0.0792	0.0446, 0.0988	0.0375, 0.0858	0.0484, 0.1138
Largest diff. peak and hole / A^{-3}	0.41, –0.40	0.46, –0.49	0.36, –0.39	0.37, –0.47
CCDC number	2476937	2476940	2476938	2476939

^{a} $R_{int} = \Sigma|F_o^2 - F_{o,mean}^2|/\Sigma F_o^2$, ^{b} $R_1 = \Sigma(||F_o| - |F_c||) / \Sigma|F_o|$; $wR_2 = [\Sigma w(F_o^2 - F_c^2)^2 / \Sigma w(F_o^2)^2]^{1/2}$

Table S2 The results of QT-AIM analysis for **1-4** using AIMALL and CAM-B3LYP + D4/ jorge-TZP-DKH calculations

1-Mn

Atoms	$\rho(\mathbf{r})$	$\nabla^2\rho(\mathbf{r})$	$h_e(\mathbf{r})$	$\lvert V(\mathbf{r})\rvert/G(\mathbf{r})$	Type of bond	DI(A|B)	Ellipticity
Mn1 – N1	0.0593	0.1852	-0.01609	1.258	coordinate	0.281	0.0155
Mn1 – N2	0.0490	0.1311	-0.01354	1.292	coordinate	0.246	0.0071
Mn1 – N3	0.0461	0.1207	-0.01240	1.291	coordinate	0.227	0.0113
Mn1 – O1	0.0451	0.1608	-0.01050	1.207	coordinate	0.205	0.0318
Mn1 – O2	0.0456	0.1615	-0.01078	1.211	coordinate	0.208	0.0358
Mn1 – O3	0.0592	0.2172	-0.01534	1.220	coordinate	0.287	0.1010
Mn1 – O4	0.0612	0.2277	-0.01588	1.218	coordinate	0.292	0.1015

2-Fe

Atoms	$\rho(\mathbf{r})$	$\nabla^2\rho(\mathbf{r})$	$h_e(\mathbf{r})$	$\lvert V(\mathbf{r})\rvert/G(\mathbf{r})$	Type of bond	DI(A|B)	Ellipticity
Fe1 – N1	0.0631	0.2582	-0.01295	1.167	coordinate	0.340	0.7667
Fe1 – N2	0.0515	0.1577	-0.01021	1.206	coordinate	0.268	0.0933
Fe1 – N3	0.0488	0.1432	-0.00936	1.207	coordinate	0.250	0.0542
Fe1 – O1	0.0413	0.1756	-0.00508	1.104	coordinate	0.204	0.3073
Fe1 – O2	0.0401	0.1727	-0.00485	1.101	coordinate	0.203	0.4408
Fe1 – O4	0.0644	0.3035	-0.01303	1.147	coordinate	0.340	0.4823
Fe1 – O3	0.0629	0.2954	-0.01251	1.145	coordinate	0.336	0.4416

3-Co

Atoms	$\rho(\mathbf{r})$	$\nabla^2\rho(\mathbf{r})$	$h_e(\mathbf{r})$	$\lvert V(\mathbf{r})\rvert/G(\mathbf{r})$	Type of bond	DI(A|B)	Ellipticity
Co1 – N1	0.0667	0.2602	-0.01392	1.176	coordinate	0.343	0.6548
Co1 – N2	0.0525	0.1834	-0.00937	1.170	coordinate	0.296	0.3456
Co1 – N3	0.0510	0.1731	-0.00883	1.169	coordinate	0.277	0.5112
Co1 – O1	0.0377	0.1651	-0.00310	1.070	coordinate	0.195	0.3086
Co1 – O2	0.0393	0.1724	-0.00360	1.077	coordinate	0.201	0.3889
Co1 – O3	0.0612	0.2953	-0.01134	1.133	coordinate	0.335	0.1338
Co1 – O4	0.0639	0.3115	-0.01243	1.138	coordinate	0.346	0.1597

4-Ni

Atoms	$\rho(\mathbf{r})$	$\nabla^2\rho(\mathbf{r})$	$h_e(\mathbf{r})$	$\lvert V(\mathbf{r})\rvert/G(\mathbf{r})$	Type of bond	DI(A|B)	Ellipticity
Ni1 – N1	0.0874	0.4067	-0.02200	1.178	coordinate	0.452	0.0855
Ni1 – N2	0.0579	0.2065	-0.01188	1.187	coordinate	0.326	0.1403
Ni1 – N3	0.0603	0.2191	-0.01280	1.189	coordinate	0.334	0.1160
Ni1 – O1	0.0245	0.0862	-0.00130	1.057	coordinate	0.109	0.2049
Ni1 – O2	0.0281	0.1018	-0.00200	1.073	coordinate	0.132	0.2563
Ni1 – O3	0.0692	0.3452	-0.01396	1.139	coordinate	0.371	0.0911
Ni1 – O4	0.0714	0.3559	-0.01485	1.143	coordinate	0.380	0.0871

Table S3 Results of continuous shape measures calculations using program Shape 2.1 for compounds **1–4**.[a]

	Mn(II)	Fe(II)	Co(II)	Ni(II)
CN=5[b]				
PP-5	1.302	1.157	1.277	1.694
vOC-5	23.465	23.828	23.430	23.098
TBPY-5	27.436	27.957	27.525	27.155
SPY-5	23.848	24.299	23.849	23.575
JTBPY-5	27.901	28.474	28.158	28.33
CN=7[c]				
HP-7	33.687	33.952	33.792	32.492
HPY-7	23.585	24.177	24.163	23.864
PBPY-7	1.108	1.012	1.097	1.650
COC-7	4.484	4.986	4.585	4.367
CTPR-7	3.561	3.855	3.503	3.015
JPBPY-7	3.743	3.403	3.570	3.504
JETPY-7	21.443	21.299	21.101	20.021

[a] The listed values correspond to the deviation between the ideal and real coordination polyhedral, the lowest values are in red color.

[b] PP-5 = pentagon, vOC-5 = vacant octahedron, TBPY-5 = trigonal bipyramid, SPY-5 = spherical square pyramid, JTBPY-5 = Johnson trigonal bipyramid J12.

[c] HP-7 = heptagon, HPY-7 = hexagonal pyramid, PBPY-7= pentagonal bipyramid, COC-7 = capped octahedron, CTPR-7 = capped trigonal prism, JPBPY-7 = Johnson pentagonal bipyramid J13, JETPY-7 = Johnson elongated triangular pyramid J7.

Table S4 Parameters of the extended one-set Debye model (eq. S1 and S2) for **3** measured at 0.2 T.

***T* /K**	**χ_T /10^-6^ cm^3^ mol^-1^**	**χ_S /10^-6^ cm^3^ mol^-1^**	***α***	***τ* /10^-3^ s**	***R*^2^**
1.9	10.57(4)	1.21(6)	0.216(9)	3.57(7)	0.99825
2.1	9.60(4)	1.09(8)	0.21(1)	2.92(8)	0.99703
2.3	8.84(4)	1.06(7)	0.18(1)	2.40(6)	0.99725
2.5	8.27(4)	1.04(8)	0.17(1)	2.02(5)	0.99671
2.7	7.63(5)	1.0(1)	0.14(2)	1.67(6)	0.99275
2.9	7.27(2)	0.96(5)	0.143(8)	1.42(2)	0.99878
3.1	6.87(3)	0.93(7)	0.13(1)	1.19(3)	0.99725
3.3	6.45(3)	0.95(9)	0.11(2)	1.0(3)	0.99592
3.5	6.15(3)	0.9(1)	0.11(2)	0.8(3)	0.99531
3.7	5.85(2)	0.91(6)	0.10(1)	0.64(1)	0.99825
3.9	5.55(1)	0.92(6)	0.08(1)	0.5(1)	0.99881
4.1	5.30(1)	0.94(5)	0.072(9)	0.40(7)	0.99925
4.3	5.08(1)	0.97(8)	0.06(1)	0.31(9)	0.99879
4.5	4.89(1)	0.98(8)	0.06(9)	0.25(8)	0.999
4.7	4.706(8)	1.02(8)	0.06(1)	0.20(7)	0.99938
4.9	4.50(2)	1.2(3)	0.03(4)	0.16(2)	0.99526
5.1	4.353(8)	1.3(1)	0.02(1)	0.134(8)	0.9993
5.3	4.207(6)	1.3(2)	0.02(1)	0.105(7)	0.99953
5.5	4.072(7)	1.4(2)	0.02(1)	0.09(1)	0.99934
5.7	3.935(6)	1.5(2)	0	0.076(4)	0.99956
5.9	3.83(2)	1(1)	0	0.06(3)	0.99677

Table S5 Calculated individual non-zero contributions to *D*-tensor for studied complexes **1**–**4** obtained from the CASSCF/NEVPT2 calculations using the effective Hamiltonian theory.

1

Block	Mult	Root	D	E
1	4	0	-0.460	-0.448
1	4	1	-0.418	0.406
1	4	2	0.177	-0.000
1	4	3	0.002	0.001
1	4	4	0.003	-0.000
1	4	5	-0.000	0.000
1	4	6	0.033	-0.000
1	4	7	-0.001	0.001
1	4	8	0.000	0.000
1	4	9	1.512	0.001
1	4	10	0.001	0.008
1	4	11	0.138	-0.014
1	4	12	-0.000	-0.000
1	4	13	-0.000	0.001
1	4	14	-0.009	0.004
1	4	15	-0.550	0.547
1	4	16	-0.527	-0.525
1	4	17	0.000	-0.000
1	4	18	-0.000	0.000
1	4	19	-0.001	-0.000
1	4	20	-0.000	0.000
1	4	21	-0.003	-0.003
1	4	22	-0.001	-0.001
1	4	23	-0.003	0.003
2	2	0	0.000	0.000
2	2	1	0.000	0.000
2	2	2	0.000	0.000
2	2	3	0.000	0.000
2	2	4	0.000	0.000
2	2	5	0.000	0.000
2	2	6	0.000	0.000
2	2	7	0.000	0.000
2	2	8	0.000	0.000
2	2	9	0.000	0.000
2	2	10	0.000	0.000
2	2	11	0.000	0.000
2	2	12	0.000	0.000
2	2	13	0.000	0.000
2	2	14	0.000	0.000
2	2	15	0.000	0.000
2	2	16	0.000	0.000
2	2	17	0.000	0.000
2	2	18	0.000	0.000
2	2	19	0.000	0.000
2	2	20	0.000	0.000
2	2	21	0.000	0.000
2	2	22	0.000	0.000
2	2	23	0.000	0.000
2	2	24	0.000	0.000
2	2	25	0.000	0.000
2	2	26	0.000	0.000
2	2	27	0.000	0.000
2	2	28	0.000	0.000
2	2	29	0.000	0.000
2	2	30	0.000	0.000
2	2	31	0.000	0.000
2	2	32	0.000	0.000
2	2	33	0.000	0.000
2	2	34	0.000	0.000
2	2	35	0.000	0.000
2	2	36	0.000	0.000
2	2	37	0.000	0.000
2	2	38	0.000	0.000
2	2	39	0.000	0.000
2	2	40	0.000	0.000
2	2	41	0.000	0.000
2	2	42	0.000	0.000
2	2	43	0.000	0.000
2	2	44	0.000	0.000

2

Block	Mult	Root	D	E
0	5	1	-9.907	-0.105
0	5	2	1.431	0.935
0	5	3	0.160	-0.282
0	5	4	1.366	-1.406
1	3	0	-1.189	-1.173
1	3	1	0.370	0.024
1	3	2	0.018	-0.002
1	3	3	-0.034	-0.055
1	3	4	0.092	0.001
1	3	5	0.220	-0.002
1	3	6	0.015	0.014
1	3	7	-0.115	-0.086
1	3	8	0.928	0.151
1	3	9	0.108	-0.003
1	3	10	1.314	0.001
1	3	11	-0.075	-0.083
1	3	12	-0.306	-0.293
1	3	13	0.083	-0.000
1	3	14	0.148	0.035
1	3	15	-0.036	-0.032
1	3	16	-0.292	-0.391
1	3	17	0.257	-0.012
1	3	18	0.062	-0.040
1	3	19	-0.010	0.011
1	3	20	-0.053	-0.054
1	3	21	-0.413	0.397
1	3	22	-0.121	-0.143
1	3	23	-0.009	0.009
1	3	24	0.006	0.002
1	3	25	0.003	0.010
1	3	26	0.011	0.002
1	3	27	-0.010	-0.011
1	3	28	0.192	-0.002
1	3	29	0.007	-0.001
1	3	30	-0.002	-0.002
1	3	31	0.126	-0.003
1	3	32	-0.192	-0.135
1	3	33	-0.109	0.091
1	3	34	-0.265	0.272
1	3	35	-0.010	-0.010
1	3	36	-0.009	0.007
1	3	37	-0.005	-0.004
1	3	38	0.002	0.003
1	3	39	-0.001	-0.004
1	3	40	0.068	0.001
1	3	41	0.000	-0.000
1	3	42	-0.003	-0.003
1	3	43	0.119	0.002
1	3	44	-0.083	-0.081
2	1	0	0.000	0.000
2	1	1	0.000	0.000
2	1	2	0.000	0.000
2	1	3	0.000	0.000
2	1	4	0.000	0.000
2	1	5	0.000	0.000
2	1	6	0.000	0.000
2	1	7	0.000	0.000
2	1	8	0.000	0.000
2	1	9	0.000	0.000
2	1	10	0.000	0.000
2	1	11	0.000	0.000
2	1	12	0.000	0.000
2	1	13	0.000	0.000
2	1	14	0.000	0.000
2	1	15	0.000	0.000
2	1	16	0.000	0.000
2	1	17	0.000	0.000
2	1	18	0.000	0.000
2	1	19	0.000	0.000

3

Block	Mult	Root	D	E
0	4	1	6.037	9.590
0	4	2	6.219	-5.892
0	4	3	9.533	-9.398
0	4	4	2.360	6.962
0	4	5	0.305	-0.296
0	4	6	0.046	0.052
0	4	7	0.005	-0.004
0	4	8	0.077	-0.047
0	4	9	0.082	0.058
1	2	0	-0.407	-0.409
1	2	1	-0.041	0.072
1	2	2	3.960	-0.249
1	2	3	-0.025	0.017
1	2	4	-0.772	-0.958
1	2	5	-0.428	0.488
1	2	6	-0.551	-0.354
1	2	7	-1.204	0.801
1	2	8	6.135	0.010
1	2	9	-0.010	0.005
1	2	10	0.016	0.001
1	2	11	0.003	0.004
1	2	12	-0.130	0.127
1	2	13	-0.147	-0.156
1	2	14	-0.166	0.189
1	2	15	-0.041	-0.084
1	2	16	-0.024	0.014
1	2	17	-0.090	-0.022
1	2	18	0.120	0.017
1	2	19	0.131	-0.034
1	2	20	0.190	-0.126
1	2	21	-0.112	-0.041
1	2	22	0.752	0.007
1	2	23	-0.063	0.147
1	2	24	-0.302	-0.279
1	2	25	-0.233	0.209
1	2	26	-0.244	0.233
1	2	27	-0.218	-0.227
1	2	28	-0.200	0.159
1	2	29	-0.075	-0.128
1	2	30	-0.012	0.009
1	2	31	-0.019	-0.036
1	2	32	-0.014	0.013
1	2	33	-0.002	0.002
1	2	34	-0.000	0.000
1	2	35	0.073	-0.001
1	2	36	0.003	-0.000
1	2	37	0.066	-0.006
1	2	38	-0.003	0.003
1	2	39	-0.010	-0.007

4

Block	Mult	Root	D	E
0	3	1	-51.865	0.198
0	3	2	21.250	-7.504
0	3	3	20.679	7.068
0	3	4	0.189	-0.038
0	3	5	0.169	-0.006
0	3	6	0.073	0.047
0	3	7	0.008	-0.000
0	3	8	0.014	0.002
0	3	9	-0.000	0.000
1	1	0	-0.000	-0.000
1	1	1	-0.000	0.000
1	1	2	15.217	-0.001
1	1	3	-6.825	6.005
1	1	4	-6.850	-5.970
1	1	5	-0.002	0.002
1	1	6	-0.065	0.024
1	1	7	-0.038	-0.010
1	1	8	-0.009	-0.000
1	1	9	-0.011	-0.011
1	1	10	-0.038	-0.001
1	1	11	1.406	0.025
1	1	12	-0.951	-0.400
1	1	13	-0.734	0.365
1	1	14	-0.000	-0.000

2	2	45	0.000	0.000	2	1	20	0.000	0.000
2	2	46	0.000	0.000	2	1	21	0.000	0.000
2	2	47	0.000	0.000	2	1	22	0.000	0.000
2	2	48	0.000	0.000	2	1	23	0.000	0.000
2	2	49	0.000	0.000	2	1	24	0.000	0.000
2	2	50	0.000	0.000	2	1	25	0.000	0.000
2	2	51	0.000	0.000	2	1	26	0.000	0.000
2	2	52	0.000	0.000	2	1	27	0.000	0.000
2	2	53	0.000	0.000	2	1	28	0.000	0.000
2	2	54	0.000	0.000	2	1	29	0.000	0.000
2	2	55	0.000	0.000	2	1	30	0.000	0.000
2	2	56	0.000	0.000	2	1	31	0.000	0.000
2	2	57	0.000	0.000	2	1	32	0.000	0.000
2	2	58	0.000	0.000	2	1	33	0.000	0.000
2	2	59	0.000	0.000	2	1	34	0.000	0.000
2	2	60	0.000	0.000	2	1	35	0.000	0.000
2	2	61	0.000	0.000	2	1	36	0.000	0.000
2	2	62	0.000	0.000	2	1	37	0.000	0.000
2	2	63	0.000	0.000	2	1	38	0.000	0.000
2	2	64	0.000	0.000	2	1	39	0.000	0.000
2	2	65	0.000	0.000	2	1	40	0.000	0.000
2	2	66	0.000	0.000	2	1	41	0.000	0.000
2	2	67	0.000	0.000	2	1	42	0.000	0.000
2	2	68	0.000	0.000	2	1	43	0.000	0.000
2	2	69	0.000	0.000	2	1	44	0.000	0.000
2	2	70	0.000	0.000	2	1	45	0.000	0.000
2	2	71	0.000	0.000	2	1	46	0.000	0.000
2	2	72	0.000	0.000	2	1	47	0.000	0.000
2	2	73	0.000	0.000	2	1	48	0.000	0.000
2	2	74	0.000	0.000	2	1	49	0.000	0.000
